# The national and provincial prevalence and non-fatal burdens of diabetes in China from 2005 to 2023 with projections of prevalence to 2050

**DOI:** 10.1186/s40779-025-00615-1

**Published:** 2025-06-02

**Authors:** Yu-Chang Zhou, Jiang-Mei Liu, Zhen-Ping Zhao, Mai-Geng Zhou, Marie Ng

**Affiliations:** 1 Department of Epidemiology, School of Public Health, Cheeloo College of Medicine, Shandong University, Jinan, 250012 China; 2 National Center for Chronic Noncommunicable Disease Control and Prevention, Chinese Center for Disease Control and Prevention, Beijing, 100050 China; 3 Yong Loo Lin School of Medicine, National University of Singapore, Singapore, 117599 Singapore

**Keywords:** Diabetes, Prevalence, Years lived with disability (YLDs), China, Subnational

## Abstract

**Background:**

China accounts for one-quarter of the world’s diabetes population, with significant subnational disparities. However, none of the available data have provided comprehensive estimates and projections at both regional and national levels in diabetes prevention and management. This study aimed to explore the temporal trends and geographical variations in the prevalence and non-fatal burden of diabetes by age and sex across China from 2005 to 2023, and to forecast diabetes prevalence through 2050.

**Methods:**

We conducted a population-based study based on the nationally representative surveys, and literature reviews. Using the DisMod-MR model and Chinese-specific disease disability weights, we estimated the non-fatal burdens of diabetes, including prevalence and years lived with disability (YLDs), across sexes, age groups, and locations. The temporal trend change was measured as the average annual percent change. The effect of the Human Development Index on burdens was assessed by applying Spearman’s rank correlation analysis. We further projected diabetes prevalence to 2050 under two scenarios, the natural trend and the effective intervention on body mass index (BMI).

**Results:**

In 2023, an estimated 233 million individuals in China were living with diabetes. Compared to 2005, the age-standardized rate (ASR) of prevalence has increased by nearly 50%, from 7.53% (95% CI 7.00–8.10%) to 13.7% (95% CI 12.6–14.8%) in 2023. The ASR of YLDs was estimated at 19.1 per 1000 population (95% CI 18.6–19.5) in 2023, compared to 10.5 per 1000 population in 2005. The ASR of prevalence and YLDs was consistently higher in males than in females. The provinces with the highest diabetes prevalence and disease burden were Beijing, Tianjin, and Shanghai. Our forecast results suggest that if existing trends continue, the prevalence of obesity will reach 29.1% (95% CI 22.2–38.2%) nationally by 2050, with some provinces in the northern region observing a prevalence of over 40%. Conversely, if effective obesity interventions were implemented, the growth in diabetes prevalence could potentially be suppressed by nearly 50%.

**Conclusions:**

The health burden and economic cost associated with diabetes are profound. There is an urgent need to scale up preventive efforts and improve population awareness to enhance disease management and achieve optimal treatment outcomes.

**Supplementary Information:**

The online version contains supplementary material available at 10.1186/s40779-025-00615-1.

## Background

China has the largest population in the world living with diabetes mellitus. According to the 2021 International Diabetes Federation (IDF) Diabetes Atlas, the number of individuals aged 20–79 years with diabetes in China was estimated to be 140.9 million, accounting for a quarter of the global diabetic population [1]. The prevalence of diabetes has risen substantially over the past 3 decades in China. The age-standardized rate (ASR) of prevalence of diabetes has doubled, increasing from 3% in 1990 to 6.2% in 2021 [2]. The increase has been particularly rapid among the population under the age of 40, with prevalence rising by over 100% in some age groups [3]. The surge in diabetes prevalence was closely tied to the rapid increase in obesity rates. The prevalence of obesity among adults increased from 7.1% in 2002 to 16.4% during 2015–2019 [4]. The lifestyles of Chinese residents experienced substantial alterations, particularly in terms of dietary habits and physical activity levels, including increased intake of processed foods and sedentary behaviour [5, 6]. Substantial geographical variations exist in the prevalence of diabetes. In general, diabetes tends to be more concentrated in developed urban areas and is less prevalent in underdeveloped rural places [7]. Moreover, the northern region consistently shows higher prevalence compared to other parts of the country [7, 8]. These subnational variations were partly driven by differences in sociodemographic factors and disparities in access to health services [8, 9], which underscore the importance of refining existing policies to address local gaps in diabetes prevention and management [10].

Diabetes affects multiple organ systems and causes a variety of vascular and non-vascular complications, such as diabetic retinopathy and diabetic nephropathy, cardiovascular diseases, as well as liver and kidney conditions [11]. In 2021, 6.47% of total years lived with disability (YLDs) in China were attributed to diabetes and high fasting plasma glucose (FPG) in general, ranking it the fourth highest risk factor for disease burden [3, 12]. The economic costs associated with diabetes were equally staggering. A recent study estimated that without immediate intervention, the total cost associated with diabetes in the country will reach $460 billion in 2030 [13].

Previous studies typically estimated diabetes prevalence using epidemiological surveys conducted in limited geographical areas (such as cities or districts) and for specific single years [14, 15]. Additionally, although some large-scale epidemiological surveys are representative of the Chinese population, they have not provided annual diabetes prevalence rates stratified by region over multiple consecutive years. Despite numerous studies on the burden of diabetes in China, none have provided comprehensive estimates and projections at both the regional and national levels. Global studies often use macro assumptions, like a universal set of disability weights (DWs), which may not fully represent the Chinese context [16, 17]. Timely evidence with geographical granularity is crucial. Building on the robust estimation framework from the Global Burden of Disease (GBD) study, we conducted updated analyses by synthesising data from multiple rounds of China Chronic Disease and Risk Factors Surveillance (CCDRFS) and published literature, utilising Chinese-specific DWs to estimate the non-fatal burdens of diabetes, including prevalence and YLDs, across China from 2005 to 2050. Chinese-specific DWs are derived from a survey conducted among over 500,000 Chinese individuals, reflecting the perception of the severity of disease sequelae among the Chinese population [17].

## Methods

### Data sources

Individual-level data on diabetes were extracted from the 6 rounds of CCDRFS conducted in 2004, 2007, 2010, 2013, 2015, and 2018 [18]. The CCDRFS study was approved by the ethical review committee of the National Center for Chronic and Noncommunicable Disease Control and Prevention, China CDC (201819). Detailed descriptions of CCDRFS are provided in Additional file 1: Methods and Table S1.

To supplement data from CCDRFS, we conducted a systematic literature search to identify additional data related to diabetes prevalence. The procedure of literature reviews was detailed in Additional file 1: Methods. The literature search yielded 7585 potentially eligible articles, and 73 articles were retained based on inclusion and exclusion criteria (Additional file 1: Methods, Table S2 and Fig. S1). In brief, all studies included in this research used population-representative samples, and only studies that explicitly mentioned diagnostic criteria were included. The diagnostic criteria include World Health Organization (WHO) 1999, American Diabetes Association (ADA) 1997, and ADA 2010. In total, the CCDRFS and literature provided 12,032 unique data points from 2001 to 2020.

The demographic information (age, sex) was from the 2010 National Population Census and China’s National Bureau of Statistics [19]. Additionally, the human development index (HDI) comprehensively reflects the health, education, and standard of living of the population in a specific region. National and provincial HDI values were obtained from the Area Database of the Global Data Lab [20]. Based on 2023 HDI values, the 31 provinces were categorised into 5 development levels.

### Estimation of prevalence

The definition of diabetes followed the 1999 WHO criteria [21]. The 1999 WHO criteria include a self-reported previous diagnosis of diabetes by medical institutions, FPG value of 7.0 mmol/L or greater, and/or a 2-hour post-load plasma glucose value of 11.1 mmol/L or greater (after a 75 g oral glucose tolerance test). Notably, participants who had been previously diagnosed with diabetes but had normal blood glucose levels at the time of the CCDRFS were still counted as having diabetes.

Diabetes prevalence for all age groups by sex and province from 2005 to 2023 was estimated using the DisMod-MR method from the GBD modelling framework by synthesising CCDRFS and literature data [12, 22]. Our estimation process for the prevalence of diabetes over multiple years involved two key steps. First, we applied the crosswalk process within the DisMod-MR model to adjust for systematic errors arising from varying diagnostic criteria, and then obtained the error-adjusted prevalence rates as input data. The crosswalk process involved an ecological comparison of diabetes prevalence estimates derived from the reference diagnostic criteria and those derived from alternative diagnostic criteria. In this process, we incorporate study-level indicators as covariates and introduce a variable to distinguish between case definitions (with reference = 0 and alternative = 1). This approach enabled us to adjust prevalence estimates according to other diagnostic standards. This error-adjusted prevalence correlated well with reference criteria from CCDRFS, as evidenced by a Pearson’s correlation coefficient of 0.76 (Additional file 1: Fig. S2). Second, we utilized the DisMod-MR method to derive the final estimates of diabetes prevalence in China and its provinces from 2005 to 2023. This approach included estimating the prevalence rates for years and regions where data were unavailable from the CCDRFS or relevant literature. Socio-economic covariates included urbanization rate, GDP per capita, education years per capita, and medical beds per capita. The main analytical procedures were as follows: 1) we performed a Bayesian meta-regression on all national-level data points, across all sexes, ages, and periods, taking into account relevant socio-economic covariates; 2) leveraging the national fit as prior information, we applied a Bayesian meta-regression with provincial random intercepts and fixed effects for socio-economic and demographic variables [22]. We obtained the prevalence rates for each 1-year group by sex, province, and year, and then calculated prevalence rates for each 5-year age group (including 0–4, 5–9, 10–14, and so forth up to over 85 years old). Details of the models can be found in Additional file 1: Methods. Regional estimates were generated by aggregating provincial estimates into 6 geographical regions (North China, Northeast China, East China, South Central China, Southwest China, and Northwest China) and 5 regions by HDI quintiles, as well as for the overall China.

### Estimation of YLDs

To quantify the non-fatal burden, we calculated YLDs for diabetes by combining the estimated prevalence of diabetes, the proportion of patients with each health state, and the corresponding DWs. Health outcomes refer to the health outcomes resulting from the sequelae of various diseases. We focused on 4 health statuses, including diabetic neuropathy, moderate vision impairment, severe vision impairment, and blindness, with health statuses rates of 60.3%, 19.3%, 5.9%, 1.1%, respectively, sourced from a national retrospective study and expert validation [23]. We assumed stable prevalence rates of neuropathy and vision impairment of varying severity levels in the diabetic population over the years. A DW represents the magnitude of health loss associated with a particular health outcome, which is multiplied by the number of people living with that outcome to compute YLDs for the outcome. DWs are rated on a scale ranging from 0 to 1, with 0 equivalent to full health and 1 a state equivalent to death. The DWs corresponding to each health state were 0.094, 0.040, 0.219, and 0.221. A more detailed description of the national survey used to assess health outcomes perceptions can be found in the previous publication [17] and Additional file 1: Methods.

To account for multiple health outcomes that refer to one individual who experiences two or more of these health outcomes simultaneously is considered to be in a state of multiple health conditions, we adopted the following analytical steps: First, we estimated the prevalence of each health state for each year-sex-age-province combination by multiplying the diabetes prevalence by the proportion of patients with each health outcomes. A micro-simulation of 40,000 individuals based on the discrete probability distribution was used to estimate the co-occurrence of health outcomes for each year-sex-age-province. In detail, the proportions for moderate, severe, and blindness are 19.1%, 5.9%, and 1.1%, respectively. For each simulated individual with diabetes, we randomly assigned 1 of these 3 health outcomes based on the aforementioned probabilities. Second, we calculated the combined DW for all individuals according to their co-occurrence of health outcomes. The combined DW for an individual with two or more health outcomes is defined as: $\text{Combined }DW=1- \prod (1-D{W}_{i})$, where $D{W}_{i}$ is the *DW* for each health outcome. Third, we estimated the DW attributable to each health outcome for each individual. This was calculated by apportioning the combined DW to each contributing health outcome in proportion to the DW of a health outcome in isolation. Fourth, we estimated the YLD rate for each year-sex-age-province by summing the YLD rate for each health outcome, calculated by the average DW attributable to each health outcome across all individuals. This procedure was repeated 1000 times to capture the uncertainty in the YLD rates. The number of YLDs for each year-sex-age-province was computed by multiplying the YLD rate by the appropriate population. A detailed description of the estimated process is provided in the Additional file 1: Methods and Fig. S3.

### Statistical analysis

To enable unbiased geographical comparisons, we calculated ASRs of prevalence and YLDs using the 2010 National Population Census as the standard population. Each reported metric was estimated as the mean of 1000 draws from its distribution, with the 95% confidence interval (CI) defined by the 2.5th and 97.5th percentiles of the ordered draws.

In addition, we calculated the annual average percentage change (AAPC) to quantify changes in prevalence and burden from 2005 to 2023. The AAPC estimates, along with their 95% CI, were calculated using a generalised linear regression model that assumes a Gaussian distribution, using Joinpoint software (version 5.0.2). Furthermore, to assess the direction and strength of the association between HDI and ASR, as well as the relationship between the AAPC of ASR and HDI from 2005 to 2023, Spearman’s rank correlation analysis was applied [24].

To forecast ASR of prevalence, we used a generalised linear mixed-effects model with a log link. HDI and body mass index (BMI) were leveraged as fixed-effect covariates, while variations within each province were captured as random effects. HDI values assumed the continuation of current trajectories, whereas the BMI values varied based on the scenarios. The values of HDI and BMI by province for 2005 and 2023 are presented in Additional file 1: Fig. S4.

All statistical procedures were performed using the R software (version 4.3.2) and Python (version 3.6.9). The significance level for all statistical tests was set at 0.05.

### Forecasting

We projected the ASR of prevalence for diabetes through 2050 under two scenarios. In the first scenario, we assumed that population BMI levels from 2023 to 2050 would continue to rise at the same annual rate observed from 2005 to 2018, both nationally and at the provincial level [25]. In the second scenario, we assumed that an effective intervention, in accordance with the WHO’s voluntary global NCD targets, would keep BMI levels stable from 2023 to 2050 [26]. The first scenario represents a reference scenario. Although it does not account for major unforeseen public health crises, it offers useful forecasts to understand the potential future trend of diabetes if no effective policy changes are implemented and population BMI levels continue to increase at historical rates. The second scenario represents an optimistic projection, designed to illustrate the potential impact of interventions that could effectively halt the rise in population BMI levels, regardless of cost or feasibility. For both scenarios, we calculated the average annual rate of change in HDI from 2020 to 2023 and assumed that this rate would continue into the future. Based on this assumption, we generated projected HDI values for the years 2024 through 2050. Additionally, we applied the relative reduction ratio to represent the potential reduction in diabetes proportion achievable through effective BMI policies compared to the progression under current trends. The relative reduction ratio is calculated as: (prevalence under the BMI intervention—prevalence under natural trend)/prevalence under natural trend × 100%.

## Results

### National trends

**Fig. 1:**
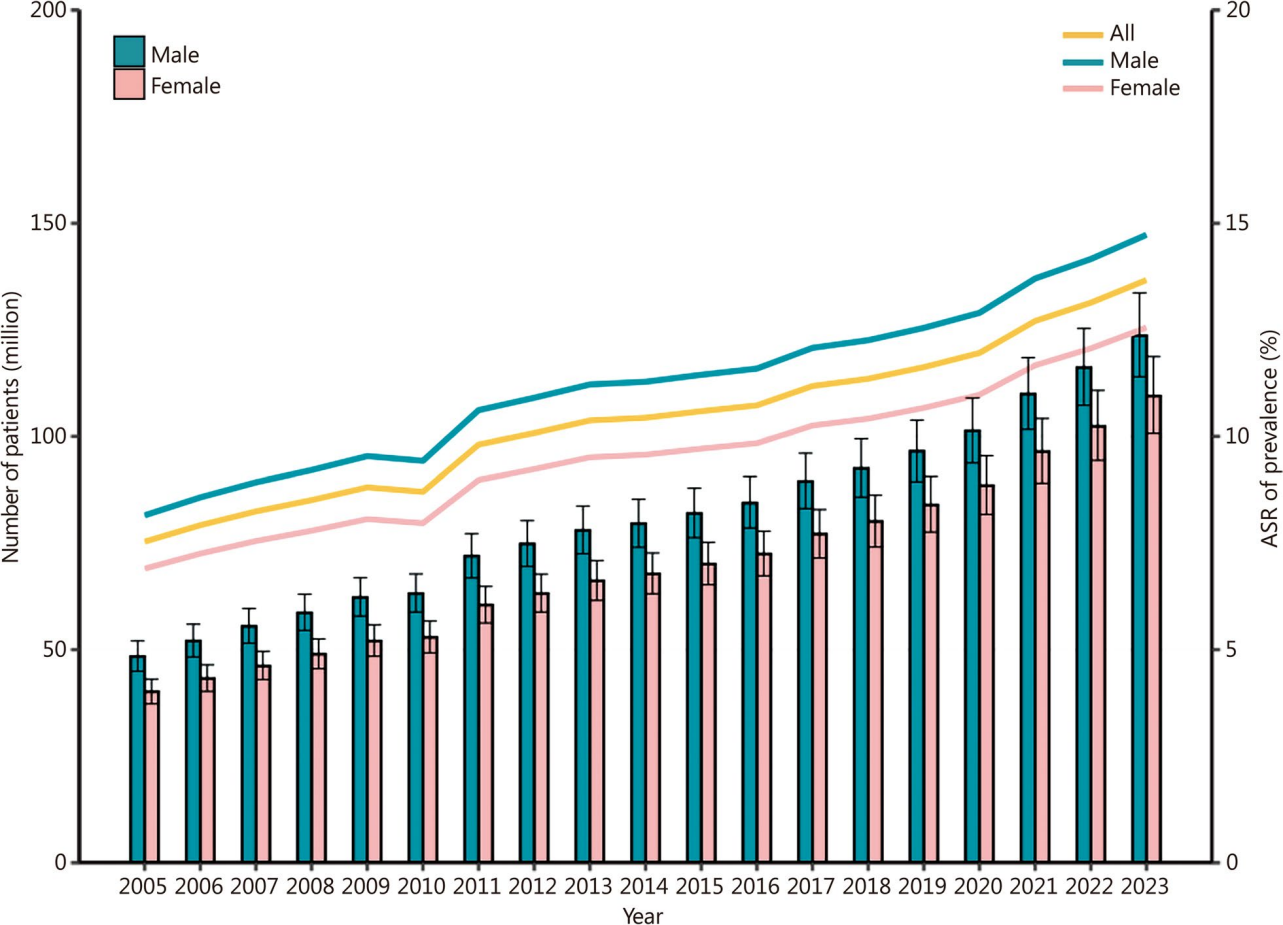
The number of patients and age-standardized rate (ASR) of prevalence for diabetes by sex in China from 2005 to 2023

In 2023, we estimated that 233.03 million (95% CI 214.65–252.37) individuals were living with diabetes in China, corresponding to a prevalence of 15.88% (Table 1). This represents a relative increase of 163.36% in total numbers compared to 88.48 million (95% CI 82.20–95.12) in 2005 (Table 1 and Additional file 1: Table S3). The ASR of prevalence rose by an average of 3.36% annually, driving ASR of prevalence from 7.53% (95% CI 7.00–8.10%) in 2005 to 13.67% (95% CI 12.59–14.80%) in 2023 (Table 1, Fig. 1 and Additional file 1: Fig. S5). The non-fatal burden, measured by YLDs, was estimated at 32.49 million (95% CI 31.75–33.24) nationally with an estimated ASR of 19.06 (95% CI 18.58–19.54) per 1000 population in 2023. Compared with the ASR of YLDs in 2005, estimated at 10.51 (95% CI 10.14–10.87) per 1000 population, the annual rate of increase averaged at 4.68% (95% CI 4.44–4.98%) (Table 1 and Additional file 1: Table S3).

**Table 1:** The number of patients, years lived with disability (YLDs), and the age-standardized rate (ASR) of prevalence and YLDs in 2005 and 2023 by sex, geographical region, and human development index (HDI), in China

Characteristics	Number of patients (million, 95% CI)		Number of YLDs (million, 95% CI)		ASR of prevalence [% (95% CI)]		ASR of YLDs [per 1000 population (95% CI)]	
	2005	2023	2005	2023	2005	2023	2005	2023
All	88.48 (82.20–95.12)	233.03 (214.65–252.37)	12.34 (11.90–12.78)	32.49 (31.75–33.24)	7.53 (7.00–8.10)	13.67 (12.59–14.80)	10.51 (10.14–10.87)	19.06 (18.58–19.54)
Sex								
Male	48.38 (44.92–52.06)	123.62 (113.96–133.66)	6.75 (6.51–6.98)	17.24 (16.85–17.63)	8.15 (7.57–8.77)	14.73 (13.58–15.93)	11.37 (10.99–11.75)	20.54 (20.04–21.03)
Female	40.11 (37.28–43.07)	109.42 (100.69–118.71)	5.59 (5.39–5.80)	15.26 (14.90–15.62)	6.90 (6.42–7.41)	12.55 (11.55–13.62)	9.62 (9.28–9.97)	17.51 (17.05–17.96)
Region								
North China	14.58 (13.52–15.63)	36.06 (33.21–38.99)	2.03 (1.97–2.10)	5.03 (4.93–5.13)	10.13 (9.40–10.86)	17.20 (15.85–18.60)	14.13 (13.71–14.55)	23.99 (23.47–24.51)
Northeast China	10.37 (9.66–11.16)	21.12 (19.47–22.84)	1.45 (1.40–1.49)	2.94 (2.88–3.00)	9.70 (9.04–10.44)	15.62 (14.40–16.89)	13.53 (13.12–13.94)	21.77 (21.27–22.29)
East China	27.53 (25.69–29.53)	78.47 (72.28–85.04)	3.84 (3.71–3.97)	10.94 (10.70–11.18)	7.65 (7.13–8.20)	14.86 (13.69–16.11)	10.66 (10.30–11.03)	20.72 (20.23–21.22)
South Central China	20.81 (19.27–22.46)	53.98 (49.71–58.49)	2.90 (2.79–3.02)	7.53 (7.33–7.73)	6.63 (6.14–7.15)	11.54 (10.62–12.50)	9.24 (8.90–9.59)	16.09 (15.65–16.54)
Southwest China	10.06 (9.30–10.81)	29.62 (27.28–32.05)	1.40 (1.34–1.46)	4.13 (4.03–4.23)	5.65 (5.23–6.08)	11.77 (10.83–12.73)	7.88 (7.56–8.20)	16.41 (15.96–16.86)
Northwest China	5.13 (4.76–5.53)	13.79 (12.71–14.96)	0.72 (0.69–0.74)	1.92 (1.87–1.97)	6.60 (6.12–7.10)	11.72 (10.81–12.72)	9.20 (8.86–9.55)	16.35 (15.90–16.79)
HDI quintile (highest to lowest)								
1	27.29 (25.45–29.31)	78.29 (72.09–84.55)	3.80 (3.68–3.93)	10.92 (10.69–11.14)	9.12 (8.51–9.80)	16.33 (15.04–17.63)	12.72 (12.32–13.12)	22.77 (22.26–23.29)
2	9.80 (9.09–10.54)	28.36 (26.17–30.80)	1.37 (1.31–1.42)	3.95 (3.86–4.05)	6.90 (6.40–7.42)	13.86 (12.79–15.05)	9.62 (9.27–9.97)	19.33 (18.85–19.82)
3	22.96 (21.28–24.71)	54.84 (50.60–59.51)	3.20 (3.08–3.32)	7.65 (7.46–7.83)	7.21 (6.69–7.76)	12.46 (11.50–13.52)	10.06 (9.70–10.42)	17.38 (16.92–17.84)
4	22.45 (20.83–24.10)	55.68 (51.20–60.39)	3.13 (3.02–3.24)	7.76 (7.59–7.94)	7.95 (7.37–8.53)	14.24 (13.09–15.44)	11.08 (10.71–11.46)	19.85 (19.36–20.34)
5	5.99 (5.55–6.47)	15.87 (14.59–17.13)	0.84 (0.79–0.88)	2.21 (2.14–2.28)	4.41 (4.08–4.76)	8.10 (7.44–8.74)	6.15 (5.86–6.44)	11.29 (10.91–11.68)

### Subnational variations

**Fig. 2:**
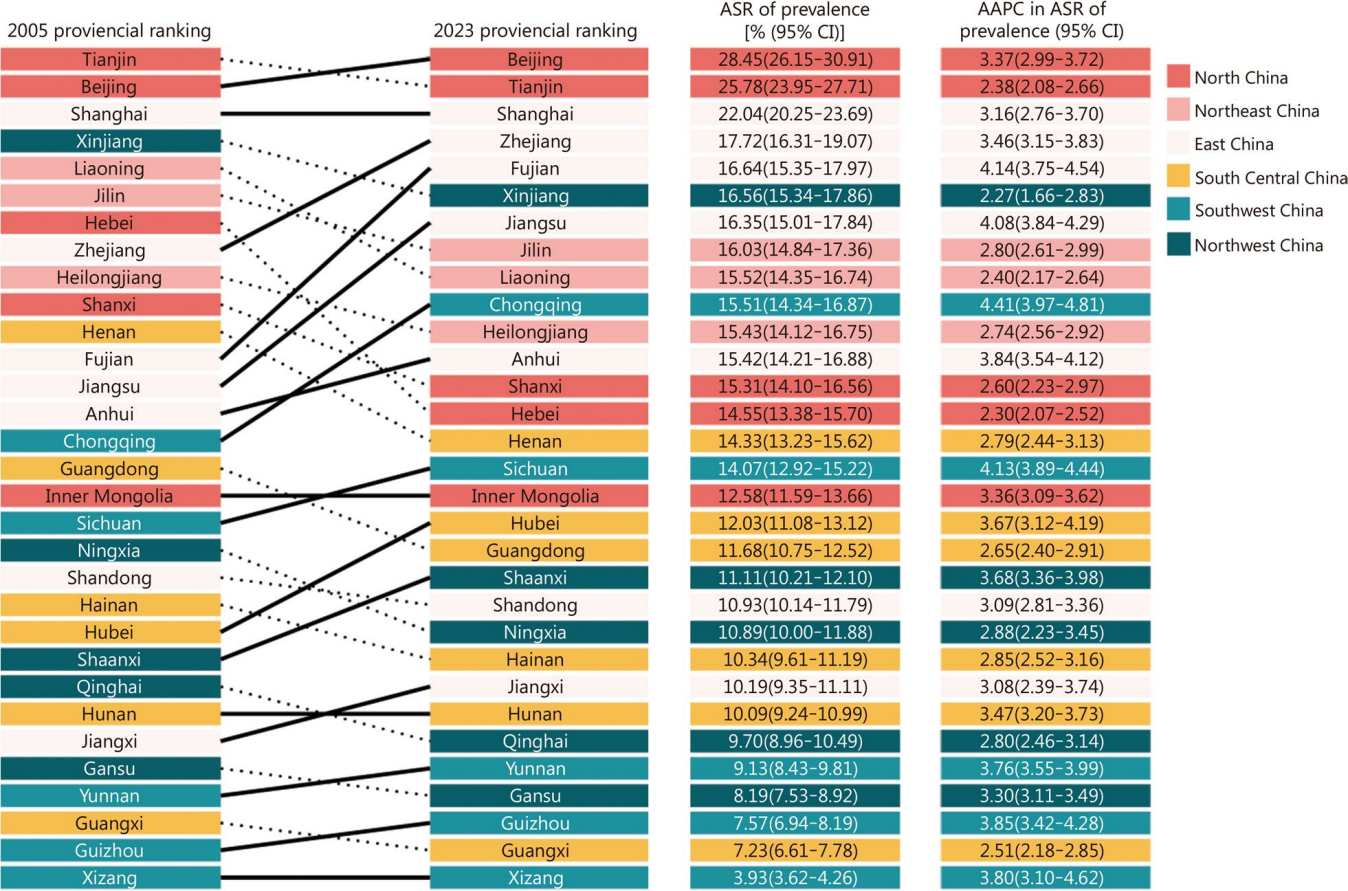
Rankings of Chinese 31 provinces in 2005 and 2023 according to the ASR of prevalence for diabetes, ASR of prevalence in 2023, and the corresponding AAPC during 2005–2023. ASR age-standardized rate, AAPC annual average percentage change

Considerable spatial variations were observed in ASR of prevalence (Table 1, Fig. 2 and Additional file 1: Table S4). In 2023, we found the largest number of individuals living with diabetes in East China, with an estimated 78.47 million (95% CI 72.28–85.04). This was followed by South Central China and North China, with an estimated 53.98 million (95% CI 49.71–58.49) and 36.06 million (95% CI 33.21–38.99), respectively (Table 1). North China had the highest ASR of prevalence, followed by Northeast China and East China, at 17.20% (95% CI 15.85–18.60%), 15.62% (95% CI 14.40–16.89%), and 14.86% (95% CI 13.69–16.11%) in 2023, respectively. Lower ASR of prevalence was observed in South Central China, Southwest China, and Northwest China, all below 12% (Table 1). Across provinces, Beijing, Tianjin, and Shanghai had the top 3 highest ASRs of prevalence, each exceeding 20%. Ten provinces observed ASR of prevalence above 15%, including Shanxi, Liaoning, Jilin, Heilongjiang, Jiangsu, Zhejiang, Anhui, Fujian, Chongqing, and Xinjiang. Xizang in Southwest China had the lowest ASR of prevalence estimated at 3.93% (95% CI 3.62–4.26%), followed by Guangxi, Guizhou, Gansu, and Yunnan, with ASRs of prevalence ranging from 7.23% (95% CI 6.61–7.78%) to 9.13% (95% CI 8.43–9.81%) (Additional file 1: Table S4).

From 2005 to 2023, ASRs of prevalence increased across all regions and provinces. The most rapid increase was observed in Southwest China, with an AAPC of 4.12 (95%CI 3.82–4.44) (Additional file 1: Table S3). ASR of prevalence rose from 5.65% (95% CI 5.23–6.08%) in 2005 to 11.77% (95% CI 10.83–12.73%) in 2023 (Table 1). In other regions, AAPCs ranged from 2.65 (95% CI 2.46–2.82) in Northeast China to 3.72 (95% CI 3.43–4.02) in East China (Additional file 1: Table S3). Across the provinces, the largest increments in ASR of prevalence were observed in Chongqing and Sichuan in Southwest China, Fujian and Jiangsu in East China, with AAPC exceeding 4% (Additional file 1: Table S4).

As for YLDs, the largest burden was observed in East China, where the total number of YLDs was estimated at 10.94 million (95% CI 10.70–11.18) in 2023, accounting for 33.67% of the national total. However, the ASR of YLDs in 2023 was highest in North China, estimated at 23.99 (95% CI 23.47–24.51) per 1000 population, and lowest in South Central China, estimated at 16.09 (95% CI 15.65–16.54) per 1000 population (Table 1). Across provinces, the highest YLD rates were observed in Beijing, Tianjin, and Shanghai, where the estimates exceeded 30 per 1000 population (Additional file 1: Fig. S6). Sharp increases in YLD rates were observed in 11 provinces, including Chongqing, Sichuan, Guizhou, Yunnan, Xizang, Shaanxi, Fujian, Anhui, Hubei, Jiangsu, and Inner Mongolia, where the AAPC exceeded 5% (Additional file 1: Fig. S6). Furthermore, similar regional variations in disease burdens of diabetes were observed across all age groups (Additional file 1: Figs. S7, S8).

### Sex and age differences

At the national level, the ASR of prevalence and YLDs for diabetes were consistently higher in males than in females (Fig. 1 and Additional file 1: Fig. S5). In 2023, the ASR of prevalence among males was 14.73% (95% CI 13.58–15.93%) and among females was 12.55% (95% CI 11.55–13.62%). These differences have remained relatively stable over time (Table 1). Conversely, the ASR of YLDs increased more rapidly in females than in males, with the AAPC being 4.85 (95% CI 4.59–5.18) for females and 4.53 (95% CI 4.31–4.81) for males during 2005–2023 (Additional file 1: Table S3).

Variations were observed between sexes across ages. In men, a marked increase in prevalence was observed between the ages of 15–19 and 20–24, where the rates leapt from 1.99% (95% CI 1.83–2.15%) to 7.42% (95% CI 6.83–8.03%). For women, the same leap was observed later between the ages of 20–24 and 25–29, with the prevalence rising from 3.54% (95% CI 3.25–3.85%) to 7.52% (95% CI 6.91–8.16%). The largest differences between males and females were in the age groups of 10–14 and 20–24, where prevalence among males was about 1.70-fold and 2.10-fold of females, respectively (Table 2). With increasing age, the sex difference in diabetes prevalence gradually narrowed and eventually stabilized. In both sexes, the prevalence continued to increase with age, reaching the highest prevalence (above 30%) in the older age groups. The prevalence ratio between males and females remained at about 1.20 from age 30 onward, and it reduced to about 1.10 from age 55 onward. A similar sex and age pattern was observed in the rates of YLDs, with males exhibiting a higher rate of YLDs in younger age groups, and gaps narrowing from age 30 onward (Table 2). Between 2005 and 2023, the largest increase in prevalence was observed in the 10–14 age group, with an AAPC of 3.88 (95% CI 3.59–4.16) (Table 2).

**Table 2:** The prevalence and rate of years lived with disability (YLDs) for diabetes in 2023, and annual average percentage change (AAPC) in the rate of prevalence and YLDs during 2005–2023, by 5-year age-sex categories in China

Age (years)	Rate of prevalence (95% CI)			Male-to-female ratio of prevalence	AAPC in prevalence (95% CI)	Rate of YLDs (95% CI)			Male-to-female ratio of rate of YLDs	AAPC in rate of YLDs (95% CI)
	Total	Male	Female			Total	Male	Female		
< 5	0.09 (0.08–0.09)	0.10 (0.09–0.11)	0.07 (0.07–0.08)	1.43	3.76 (3.47–4.05)	0.12 (0.08–0.17)	0.14 (0.09–0.19)	0.10 (0.06–0.15)	1.40	3.77 (3.50–4.06)
5–9	0.18 (0.16–0.19)	0.20 (0.18–0.22)	0.15 (0.14–0.16)	1.33	3.87 (3.59–4.15)	0.25 (0.18–0.31)	0.28 (0.21–0.35)	0.21 (0.15–0.27)	1.33	3.87 (3.62–4.17)
10–14	0.37 (0.34–0.40)	0.46 (0.43–0.50)	0.27 (0.25–0.29)	1.70	3.88 (3.59–4.16)	0.52 (0.43–0.62)	0.65 (0.54–0.76)	0.37 (0.29–0.46)	1.76	3.88 (3.64–4.17)
15–19	1.75 (1.61–1.90)	1.99 (1.83–2.15)	1.48 (1.35–1.61)	1.34	3.73 (3.45–4.00)	2.45 (2.24–2.66)	2.77 (2.55–2.99)	2.06 (1.87–2.25)	1.34	3.73 (3.48–4.00)
20–24	5.63 (5.17–6.09)	7.42 (6.83–8.03)	3.54 (3.25–3.85)	2.10	3.84 (3.59–4.08)	7.85 (7.49–8.21)	10.35 (9.94–10.77)	4.94 (4.65–5.23)	2.10	3.84 (3.62–4.09)
25–29	8.66 (7.97–9.38)	9.67 (8.91–10.46)	7.52 (6.91–8.16)	1.29	3.45 (3.18–3.72)	12.07 (11.62–12.52)	13.48 (13.01–13.95)	10.48 (10.05–10.91)	1.29	3.71 (3.45–4.04)
30–34	11.04 (10.16–11.96)	11.93 (10.99–12.89)	10.10 (9.29–10.96)	1.18	3.42 (3.15–3.68)	15.40 (14.90–15.90)	16.63 (16.12–17.15)	14.09 (13.61–14.57)	1.18	3.40 (3.13–3.70)
35–39	14.68 (13.51–15.88)	16.04 (14.78–17.33)	13.24 (12.16–14.33)	1.21	3.32 (3.05–3.58)	20.47 (19.91–21.04)	22.36 (21.78–22.95)	18.46 (17.92–19.00)	1.21	3.30 (3.04–3.60)
40–44	16.82 (15.49–18.20)	18.49 (17.04–19.97)	15.06 (13.85–16.32)	1.23	3.12 (2.84–3.41)	23.45 (22.85–24.05)	25.78 (25.16–26.40)	20.99 (20.42–21.58)	1.23	3.11 (2.84–3.41)
45–49	19.26 (17.74–20.85)	20.48 (18.88–22.14)	17.99 (16.55–19.51)	1.14	3.25 (2.90–3.54)	26.86 (26.22–27.50)	28.56 (27.91–29.22)	25.09 (24.48–25.71)	1.14	3.27 (3.00–3.59)
50–54	22.61 (20.83–24.49)	24.19 (22.30–26.15)	21.03 (19.35–22.82)	1.15	3.15 (2.83–3.42)	31.53 (30.85–32.22)	33.72 (33.03–34.43)	29.32 (28.64–29.99)	1.15	3.15 (2.93–3.42)
55–59	26.70 (24.60–28.92)	27.84 (25.69–30.12)	25.55 (23.51–27.71)	1.09	3.20 (2.86–3.48)	37.22 (36.50–37.94)	38.82 (38.09–39.56)	35.62 (34.90–36.32)	1.09	3.21 (2.98–3.48)
60–64	28.83 (26.56–31.21)	30.05 (27.70–32.47)	27.62 (25.41–29.94)	1.09	3.35 (3.01–3.63)	40.20 (39.45–40.96)	41.89 (41.13–42.67)	38.51 (37.77–39.26)	1.09	3.37 (3.11–3.68)
65–69	30.28 (27.91–32.81)	31.60 (29.14–34.17)	29.03 (26.73–31.51)	1.09	3.10 (2.83–3.38)	42.22 (41.45–42.98)	44.06 (43.27–44.83)	40.48 (39.73–41.22)	1.09	3.35 (3.11–3.65)
70–74	31.50 (29.03–34.14)	32.90 (30.33–35.58)	30.19 (27.81–32.78)	1.09	3.28 (2.92–3.58)	43.93 (43.15–44.70)	45.88 (45.10–46.67)	42.09 (41.33–42.86)	1.09	3.28 (3.05–3.57)
75–79	33.47 (30.85–36.30)	35.06 (32.34–37.95)	32.04 (29.52–34.82)	1.09	3.15 (2.79–3.44)	46.66 (45.87–47.45)	48.87 (48.07–49.67)	44.68 (43.90–45.46)	1.09	3.15 (2.91–3.44)
80–84	35.08 (32.32–38.03)	36.89 (34.02–39.93)	33.60 (30.92–36.48)	1.10	3.13 (2.78–3.41)	48.92 (48.12–49.72)	51.44 (50.62–52.26)	46.86 (46.07–47.65)	1.10	3.13 (2.90–3.41)
≥ 85	35.74 (32.90–38.73)	37.86 (34.89–40.96)	34.32 (31.56–37.23)	1.10	3.28 (2.92–3.57)	49.84 (49.04–50.64)	52.79 (51.97–53.62)	47.85 (47.06–48.65)	1.10	3.28 (3.05–3.58)

### Association of the HDI on prevalence and YLDs

A positive association was found between HDI and the prevalence, as well as HDI and YLDs. The trends and correlation of ASR of prevalence and YLDs with HDI quintiles were presented in Additional file 1: Figs. S9, S10. In general, ASR of prevalence increased with increased HDI, with a Pearson correlation of *r* = 0.70 (*P* < 0.0001). Changes in ASR of prevalence over time were moderately correlated with changes in HDI, with a Pearson correlation of *r* = 0.62 (*P* = 0.0002). The ASR of prevalence was lowest in the 1st HDI quintile (highest HDI) region, estimated at 16.33% (95% CI 15.04–17.63%), and highest in the 5th HDI quintile (lowest HDI) region, estimated at 8.10% (95% CI 7.44–8.74%) in 2023. Notably, however, the 4th HDI quintile region had the second lowest, as opposed to the highest ASR of prevalence, estimated at 14.24% (95% CI 13.09–15.44%) in 2023 (Table 1). Between 2005 and 2023, the HDI quintile with the most rapid increase in ASR of prevalence was the 2nd quintile region, with an estimated AAPC of 3.79 (95% CI 3.37–4.18) (Additional file 1: Table S3). Similarly, the ASR of YLDs also increased along with the advancing HDI, which was 11.29 (95% CI 10.91–11.68) per 1000 population, 17.38 (95% CI 16.92–17.84) per 1000 population, and 22.77 (95% CI 22.26–23.29) per 1000 population in the last, 3rd, and 1st HDI quintile region respectively in 2023 (Table 1).

### Forecasts of diabetes prevalence to 2050 under different intervention scenarios

**Fig. 3:**
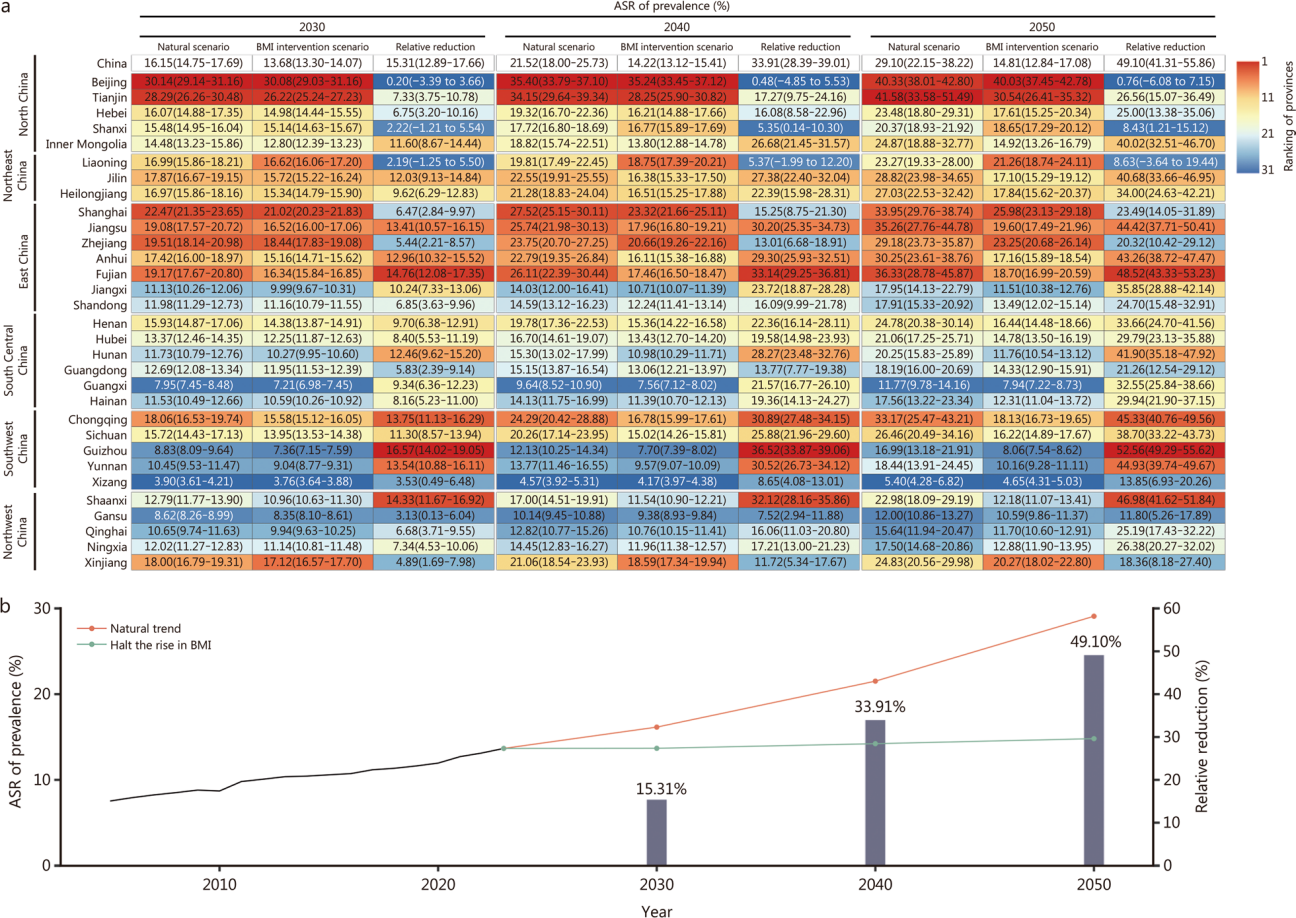
ASR of prevalence for diabetes in China during 2005–2023 (**a**), and projections of ASR of prevalence through 2050 in China and its provinces under two scenarios (**b**). ASR age-standardized rate

Assuming the existing trends persist, our forecast results suggest that the ASR of prevalence for diabetes in China will continue to increase linearly over time, reaching 16.15% (95% CI 14.75–17.69%) in 2030, 21.52% (95% CI 18.00–25.73%) in 2040, and 29.10% (95% CI 22.15–38.22%) in 2050 (Fig. 3a). Conversely, assuming specific policies are implemented to halt the rising obesity epidemic, we projected that the ASR of prevalence will gradually flatten and remain below 15% nationally in 2050. Comparing the two scenarios, the implementation of interventions targeting obesity could potentially reduce the ASR of prevalence by 15.31% (95% CI 12.89–17.66%), 33.91% (95% CI 28.39–39.01%), and 49.10% (95% CI 41.31–55.86%) in 2030, 2040, and 2050, respectively (Fig. 3b).

At the regional and provincial levels, forecast results suggest that the northern and eastern parts of China will continue to rank highest in the ASR of prevalence for diabetes during 2024–2050 (Fig. 3a). Within these regions, Tianjin and Beijing are expected to have ASR of prevalence exceeding 40%, while Fujian, Jiangsu, and Shanghai are projected to have ASR of prevalence exceeding 33% by 2050 if current trends continue. However, if BMI interventions are implemented, a slowdown in the increase in ASR of prevalence will be observed across provinces, with the most substantial impact expected in Fujian and Jiangsu, where the relative reduction in ASR of prevalence will be 48.52% (95% CI 43.33–53.23%) and 44.42% (95% CI 37.71–50.41%), respectively. In most provinces of Western China, where ASR of prevalence of diabetes has traditionally been low, our forecasts suggest that with the continuation of existing trends, ASR of prevalence in those regions will exceed 12% by 2050. In certain provinces, including Chongqing, Guizhou, and Shaanxi, the implementation of BMI interventions could potentially curb the rising diabetes trend by as much as 52% (Fig. 3a).

## Discussion

This is a comprehensive study examining the current and future prevalence and non-fatal burden of diabetes in China at the national, regional, and provincial levels. Leveraging nationally representative CCDRFS data and a systematic literature search, we estimated that 15.88% of the total population was living with diabetes in 2023. Moreover, using Chinese-specific DWs, we estimated that the non-fatal disease burden attributable to diabetes, measured by total YLDs, was 32.49 million in 2023. Without immediate intervention, nearly 1 in 3 people in China will experience diabetes by 2050. Conversely, if interventions targeting obesity were implemented, the potential increase could be reduced by half, keeping prevalence below 15% in 2050.

Our results differed from the estimates from the GBD 2021 study [1, 22, 27]. The estimated ASR of prevalence, based on the WHO 2000 world standard population, was 10.8% (95% CI 10.0–11.7%) in this study and 6.3% (95% CI 5.5–7.2%) in the GBD 2021 study, respectively [3]. There are several potential explanations for this discrepancy. First, the data sources used in the two studies were different. In GBD 2021, epidemiological data from 204 countries were obtained and analysed collectively, whereas in our study, we focused only on China-specific data, which included 6 rounds of CCDRFS and published literature. The GBD 2021 study indicated that they utilized data from the CCDRFS for the years 2010 and 2013, which may not provide a comprehensive basis for long-term trend analysis. Second, we observed that our estimates were higher than those of the GBD study in all age groups, particularly among children and adolescents. Recognizing that there is a relative scarcity of literature reporting on diabetes prevalence in Chinese children, we acknowledge that this could potentially contribute to an overestimation of diabetes prevalence in our study. Third, in terms of YLDs, the GBD study used DWs from a global sample [28], whereas in this study, we used Chinese-specific DWs. Using China-specific DWs can reflect the Chinese population’s perception of individual health states based on a specific cultural context [17]. Additionally, our estimates are relatively higher than those of the IDF 2021. We reviewed the data sources used by IDF 2021 for estimating diabetes prevalence in China, which are based on literature related to the CCDRFS. The prevalence rates reported in CCDRFS-related literature from 2010 to 2018 were calculated based on the 2010 Chinese population structure. However, from 2010 to 2020, China’s birth rate continued to decline, and aging became more severe, leading to significant changes in the population structure. As a result, the prevalence rates standardized to the 2010 population may, to some extent, be lower than the actual prevalence rates. IDF 2021 estimated that the number of people with diabetes in China in 2021 was 140 million, with a prevalence rate of 10.6%, which is also lower than the prevalence rate of 12.8% (based on the 2010 Chinese population structure) provided by the 2018 CCDRFS data. We believe these discrepancies may be related to IDF’s joint estimation of diabetes prevalence across multiple countries worldwide. Furthermore, to assess the potential overestimation in our estimates, we calculated the diabetes prevalence for Chinese adults aged 18 and above (Additional file 1: Table S5). Based on the 2020 Chinese population structure, the ASR of prevalence in 2018 was 16.40% (95% CI 15.22–17.70%) in this study, higher than that of the 2018 CCDRFS [14.83% (95% CI 12.03–15.74%)], although the difference was not statistically significant. We found that the higher prevalence estimate in the 20–39 age group in this study was the primary source of the discrepancy. Potential reasons for this include differences in sample selection between the national and local surveillance surveys incorporated into our estimates. The stratified sampling approach used in local surveys may have accounted for region-specific factors [29]. Additionally, due to the substantial workload involved in conducting national surveys, there may have been a tendency to select individuals with better health status and higher compliance in practice. We also calculated the ASR of prevalence in 2018 from the GBD 2021 study based on the 2020 Chinese population structure, which was 10.0% (95% CI 8.9–11.3%), lower than our estimate.

Since the 1990s, the prevalence of diabetes in China and India has continued to increase [15]. India ranks first in the world in terms of diabetes prevalence, exceeding 20% in 2022 [30]. The Indian population typically consumes a diet high in carbohydrates and low in protein, which can easily induce insulin resistance and elevate blood sugar levels, potentially being one of the main reasons for the high prevalence of diabetes in India [31]. The United States has the highest obesity rate in the world, which significantly contributes to its relatively high diabetes prevalence of about 12% in 2022 [30, 32]. However, despite having a higher obesity rate, the prevalence of diabetes in the United States is lower compared to China and India. This difference may be related to genetic factors [15]. Studies have shown that East Asians, including Chinese individuals, have lower β-cell function compared to Caucasians. Furthermore, at the same BMI level, Chinese individuals often have a higher percentage of body fat and more cardiovascular risk factors than Caucasians [15, 33].

The rise in diabetes reflects the rapid epidemiological shifts where non-communicable diseases now dominate mortality and disability [27]. Socio-environmental changes had altered the lifestyle of the Chinese population [34]. Studies have found a marked increase in the intake of sodium and fat [33, 35, 36]. Processed food consumption has been growing at a rate of 50% a year [37]. Meanwhile, over 20% of the population had insufficient physical activity in 2018 [38]. A study concluded that low whole-grain intake and high refined-grain intake, combined with low physical activity, each contribute to at least 20 million cases of diabetes [39]. Additionally, high smoking rates and rising obesity further exacerbate the diabetes epidemic [40, 41]. Notably, we observed a reduced diabetes prevalence rate in 2010. In 2009, China experienced an outbreak of H1N1 influenza, and in 2010, the excess mortality rate related to influenza reached 6.73 per 100,000 population [42]. One previous study has indicated that, compared to individuals without diabetes, the odds ratio (*OR*) for influenza-related mortality among diabetic patients was 8.83 (95% CI 2.04–38.20) [43]. We speculate that the increase in influenza-related deaths among diabetic patients in 2010 may have contributed to a temporary decline in the observed number of diabetic patients.

Regional disparities in diabetes burden are partly explained by socio-environmental factors. In the northern region with the highest ASR of prevalence of diabetes, the traditional dietary pattern includes a high intake of carbohydrates and low consumption of vegetables, which may increase the risks of obesity and diabetes [44, 45]. Moreover, the prevalence of physical inactivity in the northern region tends to be higher than in other regions of the country [46]. In economically developed regions, a significant transformation in the population’s diet has been driven by the remarkable expansion of the fast-food industry [47]. The formation of obesogenic and diabetogenic environments is infiltrating different parts of the countries [48], with less developed regions gradually catching up in diabetes prevalence and burden.

Genetic predisposition is another important determinant. Clinical and experimental research has demonstrated that early life experiences can impact the lifelong likelihood of developing metabolic dysfunction via epigenetic pathways [49]. Early-life exposure to the Great Chinese Famine between 1959 and 1961 is believed to have heightened the susceptibility to diabetes among cohorts born or raised during that era [50]. Additionally, genetic factors cause ethnic differences in diabetes vulnerability. Tibetans have the lowest diabetes prevalence, while Chinese Han have the highest, almost triple that of Tibetans [8, 34]. Genetic predisposition combined with socio-environmental changes has significantly increased diabetes rates in China over the past decades [51].

Consistent with previous studies [2, 9, 52], our results indicated higher diabetes prevalence and YLDs among males compared to females. This is likely driven by the disproportionately high prevalence of smoking and the rapidly increasing obesity rates in Chinese men [5, 53]. Nicotine intake from tobacco can lead to changes in blood glucose metabolism and elevated blood glucose levels, subsequently contributing to the development of diabetes [54]. Furthermore, social culture and biological features are also critical determinants. Diabetes prevalence is higher in educated males but lower in educated females [4]. Biologically, oestrogen protects women from diabetes by improving insulin sensitivity and reducing β-cell death [55]. Sex differences are more significant before 30, narrowing thereafter. An important risk factor for females is gestational diabetes (GDM) [56]. Currently, there is no national mandatory screening for GDM in China, and relatively few surveys focus on the increasing threat of GDM [57]. A meta-analysis study suggests that the total incidence of GDM in China is approximately 14.8% [57]. However, considerable heterogeneity exists across regions, and the risk elevates with maternal age [58]. A study in Southern China found that diabetes incidence among pregnant women increased from 3.03% for those under 20 to 22.40% for 30–34-year-old, and reached 42.39% among women aged 40 and above [59]. Despite China’s declining birth rate [60], the rising average maternal age indicates a significant GDM risk for diabetes [61]. Improvement in screening, monitoring, and management is particularly crucial [62]. Additionally, the elderly population exhibits a considerably higher diabetes prevalence. This may be related to medical advances reducing mortality and prolonging disease duration [63].

A major challenge in diabetes prevention and management is the lack of awareness and knowledge of the disease [9, 15, 52]. In a large nationally representative cohort study, less than a quarter of those who had diabetes were aware of the condition [9]. Poor medication adherence and self-management also resulted in higher risks of diabetes-related complications [14, 64]. This is especially true in rural China, where diabetes is rapidly increasing [52, 65]. Addressing these gaps, especially in rural areas, can help prevent new cases and improve outcomes.

Our forecast results indicate that if no intervention takes place, the prevalence of diabetes will reach 30%. This increase is driven in part by the early onset of diabetes and the rise in life expectancy [66, 67]. Obesity is a key risk factor for diabetes [41]. Controlling BMI can significantly reduce diabetes prevalence by 2050. The GBD study predicts a near doubling of YLDs by 2050 if current trends continue, with half due to high BMI. Eliminating behavioural and metabolic risk factors can lead to a significant decline in YLDs by 2050 [68].

Recently, there has been an increasing focus on the obesity epidemic in China [69]. Various policies and strategic plans, including the latest Healthy China 2030 initiative, have laid out blueprints to expand obesity prevention and management across the nation [6, 70]. The focus has shifted from lifestyle changes to addressing structural factors like food systems, built environment, healthcare, and fiscal policies [4, 6]. However, China faces unique challenges in obesity prevention due to its large population and influence of the Western food industry [47, 71]. Consumer products, such as labour-saving household appliances, sedentary lifestyle, and automobiles replacing bicycles are all influencing factors [72, 73]. In addition, poor air quality in some regions deters physical activity and exacerbates the trend of obesity and diabetes [74, 75]. Addressing these systemic forces requires significant governmental commitment and investment [6, 76]. As shown in our forecasting study, if decisive and forceful actions are taken, the payoff can be significant. Accordingly, several specific policy recommendations can be considered. First, the Chinese health administrative department should initiate public health campaigns that emphasise the importance of balanced diets and regular activity in preventing and controlling diabetes targeting men and the elderly population. Second, tailored screening programs should be established for high-risk groups, including men with obesity and residents of economically developed provinces such as Beijing and Shanghai. Third, provinces with advanced economies and a high prevalence of diabetes should reallocate funds to support the development of innovative technologies, such as digital health tools designed for early interventions of diabetes. Lastly, relevant departments in Northeastern China should enhance access to healthcare services for diabetes prevention, which include regular check-ups, educational programs, and financial support to promote health-seeking behaviour.

## Strengths and limitations

This study is unique in several ways. First, to our knowledge, this is a study to systematically estimate and forecast diabetes prevalence and non-fatal disease burden by age and sex across 6 regions and 31 provinces in China. Second, we used Chinese-specific DWs, reflecting cultural perspectives. Previous studies based on global data assumed universal standards for health outcomes that were not context specific. Finally, we provided diabetes projections under current trends and with effective obesity interventions, aiding policymakers in understanding the diabetes epidemic’s severity and potential benefits of interventions.

Our study has limitations. First, we did not distinguish type 1 and type 2 diabetes, potentially affecting targeted prevention efforts in high-prevalence provinces [77, 78]. Second, estimates for the younger population should be interpreted with caution. Data on the prevalence of diabetes in children and adolescents are relatively insufficient due to the lack of nationwide surveillance, which could potentially contribute to an overestimation of diabetes prevalence. Third, in this study, we prioritised diabetic neuropathy and vision impairment as the main sequelae for calculating YLDs due to their higher prevalence and availability of DWs in China. The proportions of patients with other sequelae are relatively lower, such as diabetic foot (approximately 5.7% of patients) [79]. Future studies can consider more sequelae for a more comprehensive assessment. Besides, our consideration of background diabetic retinopathy as causing visual impairment is somewhat arbitrary and may lead to an overestimation of the proportion of diabetes-related visual impairment. Meanwhile, previous research indicates that the prevalence of diabetic retinopathy (ranging from 24.7% to 37.5%) has remained relatively stable [80]. However, there is limited evidence on the temporal trends of diabetic neuropathy. We assumed that the prevalence rates of neuropathy and vision impairment across varying severity levels in the diabetic population have remained stable over the years. Therefore, the interpretation of YLD results should be approached with caution. Fourth, in our analysis, we chose to use socio-economic indicators, rather than lifestyle-related factors, as covariates to predict the diabetes prevalence for specific years and regions between 2005 and 2023. This decision was made due to the difficulty in obtaining comprehensive and consistent data on lifestyle-related indicators across multiple years and regions. Instead, we employed the DisMod-MR model, along with reliable regional prevalence data sourced from the CCDRFS and existing research literature, to conduct a comprehensive estimation. This approach did not solely rely on the correlation between covariates and diabetes prevalence, but rather integrated various data sources and modelling techniques to provide a more accurate and comprehensive estimate. Fifth, as an observational epidemiological studies, our analyses provide insights into the trend in diabetes prevalence and help identify potential driving factors, but cannot establish causal relationships. Our estimates, primarily based on published epidemiological data, remained generally consistent with existing literature [81]. Sixth, we do not directly estimate the contribution of obesity to diabetes prevalence and then our obesity intervention forecasting scenarios assumed a very optimistic trend, where obesity prevalence will remain at the same level as in 2023. The impact of obesity intervention takes time, and it is unlikely that any policy can immediately curb the existing obesity epidemic. Seventh, we do not model specific trajectories for the prevalence of other risk factors, which will likely influence expected changes in diabetes prevalence. However, we present the natural scenario to frame our estimates as a maximum predicted prevalence given historical trends in awareness and treatment continue, and BMI intervention scenario to highlight the best possible outcome and the potential gains that a highly effective intervention can achieve. Eighth, the national and provincial population data for 2005–2018 used in this study were obtained from the National Bureau of Statistics. For non-census years (i.e., other than 2010), the Bureau primarily relied on annual 0.1% and quinquennial 1% sampling surveys to estimate population changes. However, the relatively small sample size may still be insufficient to fully capture demographic changes or accurately track the mobile population at the national or local level. These limitations may, to some extent, affect the accuracy of the estimated diabetes prevalence in this study. Finally, this study is specific to the Chinese context, based solely on China-centric data sources and Chinese-specific DWs. Although the results may not be readily generalisable to other countries, the specificity of this work is crucial for tailoring policies most suitable for China.

## Conclusions

In conclusion, the prevalence and non-fatal burden of diabetes are increasing rapidly across China. Driven by differences in economic development, lifestyle variations, genetic predisposition, and other environmental factors, considerable heterogeneity exists across the regions. Regions and provinces with advanced economic development are observing higher prevalence and burden, while other regions of the country are also experiencing a steady rise. If no intervention is in place, diabetes prevalence will likely double by 2050. The increase in diabetes will have a profound impact on individuals’ health and quality of life, and it will incur substantial healthcare costs. There is an urgent need to implement and scale up effective strategies to prevent and manage this rapidly rising threat. This includes launching education campaign aimed to promote a balanced diet and regular physical activity, establishing tailored screening and early intervention programs for high-risk individuals and population residing in provinces with high prevalence of diabetes, and improving access to health services in remote regions.

## Supplementary Information

**Additional file 1.** Methods. **Table S1** The baseline characteristics of the Chinese adult population in six rounds of CCDRFS. **Table S2** The basic characteristic of 73 population-based studies included in the analysis. **Table S3** The percentage change of number of patients and YLDs in 2023 relative to that in 2005, and the AAPC in the ASR of prevalence and YLDs during 2005-2023 by sex, geographical region, and HDI, in China. **Table S4** The number of cases, ASR of prevalence, YLDs, and ASR of YLDs for diabetes in 2023, as well as AAPC in the ASR of prevalence and YLDs during 2005-2023, in Chinese 31 provinces (autonomous regions and municipalities). **Table S5** ASR of prevalence for diabetes among adults aged 18 and above. **Fig. S1** Systematic review flow diagram for selection of studies. **Fig. S2** Comparison of the prevalence of diabetes from a literature review and the CCDRFS (after Crosswalk process). **Fig. S3** The diagram of the estimation process of prevalence and YLDs of diabetes. **Fig. S4** The BMI and HDI in China and by province in 2005 and 2023. **Fig. S5** The number of YLDs and ASR of YLDs for diabetes by sex in China from 2005 to 2023. **Fig. S6** ASR of YLD for diabetes in China at the provincial level in 2023, and AAPC in ASR of YLDs from 2005 to 2023. **Fig. S7** The prevalence of diabetes by five-year age groups in China and Chinese six regions, 2023. **Fig. S8** The rate of YLDs by five-year age groups in China and Chinese six regions, 2023. **Fig. S9** ASR of prevalence for diabetes by quintile of HDI during 2005–2023 (a), the correlation between HDI and ASR of prevalence in 2023 (b), and the correlation between the change of HDI and AAPC in ASR of prevalence during 2005–2023 (c). **Fig. S10** ASR of YLDs for diabetes by quintile of HDI during 2005–2023 (a), the correlation between HDI and ASR of YLDs in 2023 (b), and the correlation between the change of HDI and AAPC in ASR of YLDs during 2005–2023 (c).

## Data Availability

The datasets generated during the current study will be available from the corresponding author upon reasonable request.

## Abbreviations

AAPC: Annual average percentage change
ADA: American Diabetes Association
ASR: Age-standardized rate
BMI: Body mass index
CCDRFS: China Chronic Disease and Risk Factors Surveillance
CI: Confidence interval
DWs: Disability weights
FPG: Fasting plasma glucose
GBD: Global Burden of Disease
GDM: Gestational diabetes
HDI: Human development index
IDF: International Diabetes Federation
WHO: World Health Organization
YLDs: Years lived with disability
