# Supplementary material for: The national and provincial prevalence and non-fatal burdens of diabetes in China from 2005 to 2023 with projections of prevalence to 2050
Source: Mil Med Res. 2025 Jun 2;12:28. doi: 10.1186/s40779-025-00615-1 (PMC12128495; doi:10.1186/s40779-025-00615-1)
Supplement: Supplementary file 1 — Additional file 1. Methods. Table S1 The baseline characteristics of the Chinese adult population in six rounds of CCDRFS. Table S2 The basic characteristic of 73 population-based studies included in the analysis. Table S3 The percentage change of number of patients and YLDs in 2023 relative to that in 2005, and the AAPC in the ASR of prevalence and YLDs during 2005-2023 by sex, geographical region, and HDI, in China. Table S4 The number of cases, ASR of prevalence, YLDs, and ASR of YLDs for diabetes in 2023, as well as AAPC in the ASR of prevalence and YLDs during 2005-2023, in Chinese 31 provinces (autonomous regions and municipalities). Table S5 ASR of prevalence for diabetes among adults aged 18 and above. Fig. S1 Systematic review flow diagram for selection of studies. Fig. S2 Comparison of the prevalence of diabetes from a literature review and the CCDRFS (after Crosswalk process). Fig. S3 The diagram of the estimation process of prevalence and YLDs of diabetes. Fig. S4 The BMI and HDI in China and by province in 2005 and 2023. Fig. S5 The number of YLDs and ASR of YLDs for diabetes by sex in China from 2005 to 2023. Fig. S6 ASR of YLD for diabetes in China at the provincial level in 2023, and AAPC in ASR of YLDs from 2005 to 2023. Fig. S7 The prevalence of diabetes by five-year age groups in China and Chinese six regions, 2023. Fig. S8 The rate of YLDs by five-year age groups in China and Chinese six regions, 2023. Fig. S9 ASR of prevalence for diabetes by quintile of HDI during 2005–2023 (a), the correlation between HDI and ASR of prevalence in 2023 (b), and the correlation between the change of HDI and AAPC in ASR of prevalence during 2005–2023 (c). Fig. S10 ASR of YLDs for diabetes by quintile of HDI during 2005–2023 (a), the correlation between HDI and ASR of YLDs in 2023 (b), and the correlation between the change of HDI and AAPC in ASR of YLDs during 2005–2023 (c). [file 40779_2025_615_MOESM1_ESM.pdf]

Methods

China Chronic Disease Control and Risk Factor Surveillance (CCDRFS)

Overview and study population

The CCDRFS program, initiated in 2004, is administered by the National Center for Chronic and Non-communicable Disease Control and Prevention under the Chinese Center for Disease Control and Prevention (CDC) every 3 years [1]. The CCDRFS is a nationally representative cross-sectional survey that employs a multi-stage stratified cluster random sampling method and leverages the national disease surveillance points (DSPs) system. This study utilised data from the CCDRFS collected in 2004, 2007, 2010, 2013, 2015, and 2018 [1-5]. The national DSP system covers the geographical areas of 31 provinces, municipalities, and autonomous regions in China, and expanded from 161 DSPs to 605 DSPs in 2013 [6,7]. The survey is based on a complex multistage sampling design with an effective sample size of more than 0.6 million. Over the years, survey samples have increased from 32,987 in 2004 to 184,509 in 2018 [8]. The 2004 CCDRFS was conducted in 79 DSPs selected randomly from the 161 DSPs, with sample sizes over 30,000 [1]. The 2007 and 2010 CCDRFS were based on 161 and 162 DSPs, respectively (161 DSPs plus the Second Agricultural Division of the Xinjiang Production and Construction Corps), with sample sizes exceeding 50,000 and 100,000, respectively [3]. From 2013 onwards, the CCDRFS was implemented in 302 DSPs selected randomly from the 605 DSPs system, providing morbidity data for the general population of each province, municipality, or autonomous region, with sample sizes over 180,000 [4]. Furthermore, the CCDRFS conducted in both 2004 and 2007 included participants aged 18 – 69 years old, whereas subsequent surveys conducted after 2010 had an age range starting at over 18 years old.

The ethics committee of China CDC approved the project (201819), and written informed consent were obtained from all participants.

Sample schemes

The CCDRFS applied a multi-stage stratified cluster random sampling method to obtain a nationally representative sample of the Chinese population. The sampling process included the following 4 phases. (1) Within each sampled DSP, 4 urban sub-districts or rural townships were selected through probability-proportional-to-size (PPS) sampling proportional to population size. (2) Within each urban

sub-district or rural township, 3 administrative villages or neighbourhood communities were selected using the same sampling method. (3) One residential group was selected using a cluster random sampling method from each administrative village or neighbourhood community, and 50 households were chosen using a simple random sampling method from the residential group. (4) Within each household, one permanent resident aged over 18 and who had settled in the DSP for at least 6 months was selected using a Kish selection table [7,9]. If the survey for the residents of one household was unavailable, a replacement household with a similar family structure in the same village or community was chosen, with the replacement ratio not exceeding 10% [4]. The eligibility criteria included: 1) age ≥ 18 years (restricted to 18 – 69 years in 2004 and ranging from 15 – 69 years of age in 2007); 2) having lived in the address for more than 6 months in the past 12 months; 3) not pregnant; 4) with no serious health condition or illness that would prevent the individual from participating, including intellectual disability or language disorder.

Data collection

The CCDRFS in 2004 and 2007 utilised a standardised questionnaire and physical measurements conducted through face-to-face interviews. Starting in 2010, the CCDRFS added laboratory testing for blood samples [9,10]. The questionnaire covered demographic characteristics, alcohol consumption, smoking, dietary habits, physical activities, socioeconomic status, and history of chronic diseases, including diabetes and hypertension. Physical measurements included height, weight, waist and hip circumference, and blood pressure, etc. [10,11]. The laboratory tests for diabetes involved fasting plasma glucose (FPG) and a 2-hour post-load plasma glucose test after a 75 g oral glucose tolerance test (OGTT 2 h PG) [7,12]. Blood samples were collected from all participants who had fasted for at least 10 h. FPG was measured locally within 1 d using glucose hexokinase or oxidase methods. The 75 g oral glucose tolerance test was conducted only for participants who did not report a history of diabetes, and blood samples were collected at 0 and 2 h after oral administration. Blood specimens were collected in vacuum tubes containing anticoagulants and centrifuged within 2 h [7,12]. All these procedures followed a standard protocol.

Statistical analysis

Based on a complex sampling design, weights for prevalence were calculated [9-11]. The weights consisted of sampling weights and post-stratification weights. Sampling weights addressed unequal

probabilities of sample selection, while post-stratification weights harmonized the sample structure of the survey with that of the Sixth National Population Census. Gender, age (in 5-year increments), place of residence (urban/rural), and provinces were used as stratification variables to compute post-stratification weights. The weighted prevalence represents the overall Chinese adult population. Given the complex sampling design, standard errors were estimated using the Taylor series linearisation method with finite population correction to obtain 95% confidence intervals (CIs). Self-reported prevalence of diabetes was obtained from the 2004 and 2007 CCDRFS [1,2]. The prevalence of diabetes from the 2010 CCDRFS and beyond was based on biometric measurements and computed using World Health Organization (WHO) 1999 diagnostic criteria [3,7]. The prevalence and its 95% CI were computed for the whole population and among subgroups of age, gender, and administrative region. All analyses were conducted using SAS version 9.4 (SAS Institute Inc., Cary, North Carolina).

Literature search

The literature search was conducted in the China National Knowledge Infrastructure (CNKI), the Wanfang digital database, PubMed, and Web of Science to acquire Chinese-language and English-language publications from January 2005 to December 2023. The Medical Subject Headings (MeSH) terms and keywords included "diabetes" "prevalence" and "China" (or "Chinese"). Two authors independently performed this literature search to ensure adherence to the quality standards for reporting meta-analyses [13]. The inclusion and exclusion criteria for the literature search were as follows. For the systematic literature search, studies were deemed eligible for inclusion in this analysis if they met the following criteria: 1) nationally or regionally representative population-based observational studies published in peer-reviewed journals; 2) studies conducted in China, excluding Hong Kong, Macao, and Taiwan; and 3) studies employed random sampling methods. Studies were excluded if: 1) the studies did not report the prevalence of diabetes; 2) diagnostic criteria for diabetes were not clearly defined; 3) the studies were not based on the general population, such as those focused on specific occupational groups, ethnic minority groups, or hospital inpatients; 4) the studies only reported pre-diabetes or gestational diabetes; 5) duplication of studies or the same data appearing in multiple studies; and 6) reviews, editorials, letters, or comments.

DisMod-MR

Likelihood estimation

The following information is from the supplementary materials of the Global Burden of Disease (GBD) 2021 article [14].

There are 4 choices of likelihood functions in DisMod-MR: Gaussian, Log-Gaussian, Laplace, and Log-Laplace [15]. The default Log-Gaussian likelihood function is as follows:

$$-log\left[p\left(\left(y_j|\Phi\right)\right)\right] = log(\sqrt{2\pi}) + log(\delta_j + s_j) + \frac{1}{2}\left(\frac{log(a_j + \eta_j) - log(m_j + \eta_j)}{\delta_j + s_j}\right)^2$$

Where y_j is the measured value for data point j; Φ refers to all model random variables; η_j denotes the offset value for a particular integrand (prevalence, incidence, mortality, remission, etc.); a_j is the adjusted measured value for data point j.

$$a_j = e^{(-u_j - c_j)} y_j$$

where, u_j denotes the total area effects (i.e., the sum of the random effects at two levels of the cascade: province and country); c_j denotes the total covariate effects (i.e., the mean combined fixed effects for study covariates and sex).

$$c_j = \sum_{k=0}^{K[I(J)]-1} \beta_{I(J),k} \hat{X}_{k,j}$$

s_j is the standard deviation (SD) of c_j.

$$s_j = \sum_{l=0}^{L[I(j)-1]} \zeta_{I(J),l} \hat{Z}_{k,j}$$

where k (x-covariate) and l (z-covariate) separately denote the mean value and SD of each data point related to a covariate; $I(j)$ refers to data point j for a particular integrand; $\beta_{I(J),k}$ and $\zeta_{I(J),l}$ are the multipliers of the kth x-covariate and l^{th} z-covariate for the ith integrand respectively. $\hat{X}_{k,j}$ and $\hat{Z}_{k,j}$ are respectively the covariate value and SD of the covariate value corresponding to data point j for covariate k.

δ_j is the SD of a_j, defined by:

$$\delta_j = log\left[y_j + e^{(-u_j - c_j)}\eta_j + c_j\right] - log\left[y_j + e^{(-u_j - c_j)}\eta_j\right]$$

m_j denotes the model for the measured value of data point j, not counting measurement noise, defined

by:

$$m_j = \frac{1}{B(j) - A(j)} \int_{A(j)}^{B(j)} I_j(a)da$$

where $A(j)$ and $B(j)$ is the lower and upper bound of the age range for data point j respectively; I_j refers to the function of age corresponding to the integrand for data point j.

Modelling strategy

(1) We defined the hierarchical relationship for China, which encompasses 31 provinces. (2) The model parameters were set as follows: we assigned a prior range of 0 to 1 for prevalence aged 0 to 101. We predetermined the age knots at 20 and 75 for prevalence aged 0 to 101. We assigned prior values for the presence of moderate heterogeneity in prevalence and a smoothing coefficient of 0.002. We specified a prior distribution assuming that the prevalence rises within the 20 – 75 age bracket and subsequently declines within the 75 – 100 age bracket. The following are the estimated coefficients of the model parameters.

Study covariate	Parameter	β (95% CI)	Exponentiated β (95% CI)
Urbanization rate	Prevalence	0.015 (0.013 – 0.017)	1.015 (1.013 – 1.017)
GDP per capita	Prevalence	< 0.001 (< 0.001 – < 0.001)	1.000 (1.000 – 1.000)
Education years per capita	Prevalence	0.053 (0.049 – 0.056)	1.054 (1.050 – 1.057)
Medical beds per capita	Prevalence	0.001 (0.001 – 0.002)	1.001 (1.000 – 1.002)

The national survey on health states perceptions

Study design

This study conducted a web-based survey between May 12 and July 22, 2020, using the pair-wise comparison (PC) and population health equivalence (PHE) methods [16]. Each PC question presented a random pair of health states with a brief description of the main features in lay language and asked respondents which person they thought was healthier. The PHE questions asked respondents to compare the health benefits of two hypothetical life-saving or health-improving programs and choose which health program they thought produced the greater overall population health benefit. Annual average percentage change (AAPC) question includes two descriptions of hypothetical people, each living in a particular randomly chosen health state. For example. the PC questions were phrased as follows: “the first person has mild tremors and moves a litter slowly, but can walk and do daily activities without assistance; the second person has some trouble remembering recent events, and finds

it hard to concentrate and make decisions and plans". The respondents would select the person thy regarded as being healthier. The PHE questions were phrased as follows: "the first health program prevented 1000 population from getting an illness that causes rapid death; the second health program prevented x people from getting an illness that is nonfatal but causes the lifelong health problems of y", where x is a randomly assigned "bid" value of 1500, 2000, 3000, 5000, or 10,000 and y a lay description of a randomly chosen health state.

The derivation process of disability weights

The derivation process of disability weights comprises 3 steps. (1) Analysis of PC using a probit regression model. The implication of this is that the probit regression yields estimates of values for each health state that capture the relative differences in health levels between states, consistent with the PC responses, but that these health-state values are on an arbitrary scale. (2) Analysis of PHE. This study modelled responses to PHE questions using interval regression, which provided information on trade-offs between mortality and nonfatal outcomes. (3) Anchoring the resulting estimates of the probit regression on the 0 – 1 DW scale. This study ran an interval, linear regression on the probit coefficients of the disability weight estimates implied by the PHE responses

Six-region geographical divisions and human development index (HDI) quintiles

The 6 geographical regions of China are defined as North China, Northeast China, East China, South Central China, Southwest China, and Northwest China. North China includes 5 provinces (Beijing, Tianjin, Hebei, Shanxi, and Inner Mongolia); Northeast China includes 3 provinces (Liaoning, Jilin, and Heilongjiang); East China includes 7 provinces (Shanghai, Jiangsu, Zhejiang, Anhui, Fujian, Jiangxi, and Shandong); South Central China includes 6 provinces (Henan, Hubei, Hunan, Guangdong, Guangxi, and Hainan); Southwest China includes 5 provinces (Chongqing, Sichuan, Guizhou, Yunnan, and Xizang); Northwest China includes 5 provinces (Shaanxi, Gansu, Qinghai, Ningxia, and Xinjiang) [17].

The first HDI quintile region includes 7 provinces: Gansu, Guangxi, Guizhou, Ningxia, Qinghai, Xizang, and Yunnan. The second HDI quintile region contains 6 provinces: Anhui, Hebei, Heilongjiang, Jiangxi, Sichuan, and Xinjiang. Six provinces in the third HDI quintile region are Henan, Hunan, Jilin, Shaanxi, Shandong, and Shanxi. The 4th HDI quintile region comprises 5 provinces: Chongqing, Fujian, Hainan, Hubei, and Inner Mongolia. The 7 provinces in the top HDI quintile region, which are

relatively economically developed, include Beijing, Guangdong, Jiangsu, Liaoning, Shanghai, Tianjin, and Zhejiang.

Simulation of comorbidity correction

Given the co-existence of the sequelae, simulation analyses were performed to ensure the accurate estimation of YLDs. For each year-gender-age-region, one simulation was conducted as follows:[15]

Step 1: A simulation of 40,000 individuals with any of the 4 health states was generated. The probability of occurrence for each health state was considered independent and was calculated as the prevalence multiplied by the proportion of patients with each health state. Subsequently, the health states of each individual was determined based on a draw from the discrete probability distribution.

Step 2: For each individual, the DW was estimated based on the following according to the health states the individual experienced.

$$Individual\ DW_l = 1 - \prod_{k=i}^{j}(1 - DW_k)$$

where DW_k refers to the DW for k-th ($k = 1, 2, 3, 4$) health state that the individual $i, i = 1, 2, 3, \ldots, 40{,}000$) experienced.

Step 3: For each individual, the DW attributable to each health state was calculated by:

$$ADW_{lk} = \frac{DW_k}{\sum_{k=i}^{k=j} DW_k} \times Individual\ DW_l \tag{2}$$

Where ADW_{lk} is the attributable DW for the individual i with k-th health state.

Step 4: YLD rate for each health state was computed by the sum of the attributable DW across all individuals.

$$YLD\ rate_k = \frac{\sum_{l=1}^{n} ADW_{lk}}{n}$$

where $YLD\ rate_k$ refers to YLD per capita for k-th health state.

For each health state, the number of YLDs was calculated by YLD rate times the corresponding population, and the number of YLDs of diabetes was the sum of these.

Table S1 The baseline characteristics of the Chinese adult population in six rounds of China Chronic Disease and Risk Factors Surveillance (CCDRFS)

Year	Overall population	Weighted population (million)	Sex (%)		Sex (in weighted population) (%)		Age groups (%)			Age groups (in weighted population) (%)				Regions[a] (%)			Age-standardized prevalence rate[b] (%)	Self-reported age-standardized prevalence rate (%)
			Male	Female	Male	Female	18 – 44 years	45 – 59 years	≥ 60 years	18 – 44 years	45 – 59 years	60 – 69 years	≥ 70 years	Eastern China	Central China	Western China		
2004	32,987	822.62	44.50	55.50	51.38	48.62	52.50	34.72	12.78	64.32	26.32	9.36	-	31.50	30.76	37.74	-	1.11
2007	50,717	906.95	47.22	52.78	51.01	48.99	52.04	33.88	14.08	63.76	26.67	9.58	-	33.47	31.53	35.00	-	1.50
2010	98,658	1039.41	45.74	54.26	50.50	49.50	45.79	33.98	20.23	57.62	25.39	9.55	7.44	33.62	31.17	35.21	9.79	3.51
2013	176,534	1040.44	42.75	57.25	50.50	49.50	32.23	38.71	29.06	57.63	25.38	9.55	7.44	37.64	28.70	33.66	10.90	3.89
2015	179,514	1051.55	46.97	53.03	50.53	49.47	30.92	36.66	32.42	57.85	25.26	9.49	7.40	37.21	28.33	34.46	11.20	2.92
2018	184,509	1051.55	44.40	55.60	50.32	49.68	22.34	37.32	40.34	57.85	25.26	9.49	7.40	37.16	28.79	34.05	12.46	4.31

[a]Three-region geographical divisions of China include Eastern China, Central China, and Western China. Eastern China includes 11 provinces, Beijing, Tianjin, Liaoning, Shandong, Hebei, Jiangsu, Zhejiang, Shanghai, Fujian, Guangdong, and Hainan; Central China contains 8 provinces, Jilin, Heilongjiang, Shanxi, Henan, Anhui, Jiangxi, Hunan, and Hubei; Western China is composed of 12 provinces, Inner Mongolia, Ningxia, Gansu, Qinghai, Xinjiang, Xizang, Guizhou, Shaanxi, Chongqing, Sichuan, Yunnan, Guangxi.

[b]Age-standardized prevalence rate of diagnosed diabetes by World Health Organization (WHO) 1999 was calculated using the demographic structure of the sixth Chinese population census

Table S2 The basic characteristic of 73 population-based studies included in the analysis

First author	Years of publication	Survey period	Area	Urban/Rural	Age range (years)	Sample (*n*)	Male (%)	Diagnostic criteria†	Prevalence (%)	Reference
Guo et al.	2019	2015	China	Urban and Rural	≥ 45	15,246	47.13	WHO 1999	10.60	[18]
Chi et al.	2018	2010 – 2012	China	Urban and Rural	7 – 17	25,376	50.76	ADA 1997	0.24*	[19]
Liu et al.	2010	August 2002 to December 2004	China	Urban and Rural	≥ 18	52,416	46.60	WHO 1999	2.49*	[20]
Bragg et al.	2017	2004 – 2008	China	Urban and Rural	35 – 74	512,869	-	WHO 1999	5.90	[21]
Liu et al.	2016	June 2011 to Marth 2012	China	Urban and Rural	≥ 45	11,847	-	ADA 2010	15.80	[22]
Wu et al.	2016	2007 - 2011	China	Urban and Rural	≥ 18	23,010	46.94	WHO 1999	4.30*	[23]
Bu et al.	2015	June 2007 to May 2008	China	Urban and Rural	≥ 30	8352	100.00	WHO 1999	8.07	[24]
Yang et al.	2010	June 2007 to May 2008	China	Urban and Rural	≥ 20	46,239	39.83	WHO 1999	9.70*	[25]
Wang et al.	2015	2010	China	Urban and Rural	≥ 18	98,658	45.76	ADA 2010	11.60*	[26]
Mezuk et al.	2013	2004 – 2008	China	Urban and Rural	30 – 79	504,548	-	ADA 2010	5.30	[27]
Attard et al.	2012	2009	China	Urban and Rural	18 – 90	7741	-	WHO 1999	12.00	[28]
Li et al.	2020	2015 – 2017	China	Urban and Rural	≥ 18	80,937	50.55	WHO 1999	11.20	[29]
Wang et al.	2021	2018	China	Urban and Rural	≥ 18	173,642	44.19	WHO 1999	12.40	[30]
Bai et al.	2021	February 2013 to June 2013	China	Urban and Rural	≥ 45	12,458	47.27	ADA 2010	13.21	[31]
Jin et al.	2023	2017	China	Urban and Rural	≥ 20	73,340	50.20	WHO 1999	11.70	[32]
Fang et al.	2019	2014	Beijing	Urban and Rural	40 – 79	11,889	45.29	WHO 1999	12.11	[33]
Wang et al.	2021	2017	Beijing	Urban and Rural	0 – 101	2,104,159	51.36	WHO 1999	6.60*	[34]
Cao et al.	2007	Marth to October 2004	Beijing	Urban and Rural	6 – 18	19,112	49.78	WHO 1999	0.57*	[35]
Zhan et al.	2010	May to August 2007	Beijing	Urban and Rural	≥ 20	10,054	36.67	WHO 1999	10.99*	[36]
Wei et al.	2010	June to September 2007	Beijing	Urban and Rural	≥ 20	5465	-	WHO 1999	11.94*	[37]
Zhang et al.	2007	September to November 2005	Beijing	Urban and Rural	18 – 92	16,658	39.65	WHO 1999	8.80*	[38]
Liu et al.	2016	2001 and 2009 – 2010	Beijing	Urban	60 – 95	2277/2102	41.41 and 40.34	WHO 1999	21.40/24.80	[39]
Zhi et al.	2009	June to September 2005	Tianjin	Urban and Rural	15 – 74	21,454	-	WHO 1999	6.07	[40]
Chang et al.	2011	2006	Tianjin	Urban and Rural	≥ 15	2886	47.37	WHO 1999	8.14	[41]
Qian et al.	2009	Marth to April 2008	Tianjin	Urban	≥ 50	3572	43.90	ADA 1997	17.60	[42]
Li et al.	2009	2006	Tianjin	Urban and Rural	≥ 18	2885	47.38	WHO 1999	8.10	[43]
You et al.	2018	July to September 2014	Liaoning	Urban and Rural	18 – 101	17,857	48.97	WHO 1999	13.00*	[44]

First author	Years of publication	Survey period	Area	Urban/Rural	Age range (years)	Sample (*n*)	Male (%)	Diagnostic criteria†	Prevalence (%)	Reference
You et al.	2018	May to September 2016	Liaoning	Urban	≥ 18	30,953	49.16	WHO 1999	9.48*	[45]
Yang et al.	2018	April to December 2009	Liaoning	Urban	18 – 74	15,477	45.68	ADA 2010	10.90	[46]
Pan et al.	2020	October 2015 to November 2018	Liaoning	Urban and Rural	35 – 75	53,497	46.67	WHO 1999	17.96	[47]
Su et al.	2016	June to October 2012	Jilin	Urban and Rural	18 – 79	21,435	48.22	WHO 1999	9.10	[48]
Yu et al.	2016	2012	Jilin	Urban and Rural	18 – 79	16,834	45.92	ADA 2010	8.20*	[49]
Xing et al.	2021	September 2017 to March 2019	Heilongjiang	Urban and Rural	≥ 40	18,796	50.20	ADA 2010	17.10	[50]
Zhao et al.	2009	August 2005 to August 2006	Shanxi	Urban and Rural	≥ 15	12,111	43.96	WHO 1999	5.27	[51]
He et al.	2022	2017 – 2019	Shanxi	Urban and Rural	≥ 15	14,137	-	WHO 1999	9.84	[52]
Yang et al.	2017	June 2013	Anhui	Urban and Rural	≥ 18	7128	56.86	WHO 1999	11.80	[53]
Ding et al.	2016	November 2013 to May 2014	Chongqing	Urban and Rural	≥ 18	5394	37.76	WHO 1999	15.74	[54]
Wang et al.	2009	2008	Chongqing	Urban	≥ 20	3668	55.94	WHO 1999	7.44*	[55]
Gao et al.	2014	Marth to May 2012	Henan	Urban and Rural	15 – 74	18,772	44.86	WHO 1999	9.21	[56]
Tian et al.	2009	August 2007 to May 2008	Henan	Urban and Rural	29 – 74	3,300	43.58	WHO 1999	13.50	[57]
Li et al.	2022	2015 – 2019	Henan	Urban and Rural	≥ 60	45,488	42.15	WHO 1999	29.43	[58]
Zhu et al.	2013	2007	Gansu	Urban and Rural	≥ 35	2233	-	WHO 1999	5.70	[59]
Hao et al.	2015	2013	Gansu	Urban and Rural	20 – 74	31,417	44.83	WHO 1999	10.60	[60]
Chen et al.	2012	2011	Shandong	Urban and Rural	18 – 69	15,350	50.05	WHO 1999	5.50	[61]
Liang et al.	2014	February to Marth 2012	Shandong	Urban	40 – 90	8239	33.29	WHO 1999	10.70	[62]
Lyu et al.	2020	2019	Shandong	Rural	0 – 101	2168	46.60	WHO 1999	24.60	[63]
Wei et al.	2021	February 2020 to December 2020	Shandong	Rural	≥ 60	3587	42.26	WHO 1999	13.50*	[64]
Yan et al.	2018	April 2016	Jiangxi	Urban and Rural	≥ 40	625	44.00	WHO 1999	11.14	[65]
Liu et al.	2016	April to August 2015	Jiangxi	Urban and Rural	18 – 79	2580	51.24	WHO 1999	9.50	[66]
Liu et al.	2011	2010	Jiangxi	Urban and Rural	≥ 18	3000	47.83	WHO 1999	7.63	[67]
Chen et al.	2017	July 2013	Fujian	Urban and Rural	18 – 29	5851	43.72	WHO 1999	8.44*	[68]
Ye et al.	2016	October 2010 to December 2011	Fujian	Urban and Rural	≥ 18	6016	41.74	WHO 1999	10.60	[69]
Lin et al.	2009	July 2007 to May 2008	Fujian	Urban and Rural	20 – 74	3208	38.97	WHO 1999	9.51	[70]
Wang et al.	2018	Marth to June 2016	Jiangsu	Urban and Rural	18 – 65	7689	43.96	WHO 1999	6.52*	[71]
Xie et al.	2019	2014	Jiangsu	Urban and Rural	≥ 18	8204	45.01	WHO 1999	11.00	[72]

First author	Years of publication	Survey period	Area	Urban/Rural	Age range (years)	Sample (*n*)	Male (%)	Diagnostic criteria†	Prevalence (%)	Reference
Zhang et al.	2012	October to December 2010	Guangdong	Urban and Rural	≥ 18	3590	45.15	ADA 2010	21.70*	[73]
LI et al.	2012	2009	Shanghai	Urban and Rural	≥ 35	7423	46.63	ADA 1997	7.40*	[74]
Zhang et al.	2018	December 2016 to April 2017	Shanghai	Urban	18 – 65	4028	43.30	ADA 2010	8.50	[75]
Qin et al.	2016	April 2007 to January 2008	Shanghai	Urban and Rural	18 – 80	3136	44.42	ADA 2010	15.91	[76]
Tang et al.	2014	2011 - 2012	Shanghai	Urban and Rural	30 – 80	2092	33.70	ADA 2010	21.33	[77]
Yang et al.	2013	Marth to August 2010	Shanghai	Urban	40 – 92	5824	40.30	WHO 1999	21.90	[78]
Peng et al.	2023	2017	Shanghai	Urban and Rural	≥ 35	18,960	40.02	WHO 1999	19.40*	[79]
Han et al.	2019	2015 – 2016	Hubei	Urban and Rural	35 – 75	64,151	36.29	ADA 1997	22.20	[80]
Xu et al.	2013	June 2007 to May 2008	Shaanxi	Urban and Rural	≥ 20	3254	41.98	WHO 1999	8.00*	[81]
Zhang et al.	2022	2018	Sichuan	Urban and Rural	≥ 18	7817	43.64	WHO 1999	12.94	[82]
Ding et al.	2021	2018	Chongqing	Urban and Rural	≥ 15	5145	-	WHO 1999	17.90	[83]
Duan et al.	2022	2015 – 2020	Inner Mongolia	Urban and Rural	≥ 35	11,361	40.52	ADA 2010	17.20	[84]
Wei et al.	2013	June 2009 to May 2010	Qinghai	Urban and Rural	≥ 18	1574	-	WHO 1999	4.29	[85]
Ma et al.	2015	October 2010	Qinghai	Urban and Rural	≥ 18	1674	44.15	WHO 1999	4.90	[86]
Liu et al.	2019	2010 – 2012	Guizhou	Urban and Rural	≥ 18	3073	44.32	WHO 1999	4.90	[87]
Bai et al.	2019	November 2015 to June 2016	Xizang	Urban and Rural	≥ 18	2449	43.85	WHO 1999	3.24	[88]
Fu et al.	2017	2013 – 2014	Hainan	Urban and Rural	≥ 18	4697	43.70	WHO 1999	12.00	[89]
Liu et al.	2023	2014 – 2016	Hainan	Urban and Rural	≥ 100	1002	18.00	WHO 1999	9.50	[90]

*Age-standardized prevalence rate, was calculated on the basis of the weights from the standard population.

†The World Health Organization (WHO) 1999 diagnostic criteria of diabetes include fasting plasma glucose (FPG) value of 7.0 mmol/L or greater, and /or 2-hour post-load plasma glucose value of 11.1 mmol/L or greater (after a 75 g oral glucose tolerance test) (OGTT 2 h PG). The 1997 diagnostic criteria of diabetes from American Diabetes Association (ADA) contain clinical symptoms of diabetes plus random plasma glucose concentration of 11.1 mmol/L or greater, and/or FPG value of 7.0 mmol/L or greater, and/or OGTT 2 h PG level of 11.1 mmol/L or greater. The ADA 2010 diagnostic criteria of diabetes include clinical symptoms of diabetes, and/or FPG value ≥ 7.0 mmol/L, and/or OGTT 2 h PG value ≥ 11.1 mmol/L, and/or glycosylated hemoglobin (HbA1c) value ≥ 6.5%

Table S3 The percentage change of number of patients and years lost due to disability (YLDs) in 2023 relative to that in 2005, and the annual average percentage change (AAPC) in the age-standardized rate (ASR) of prevalence and YLDs during 2005 – 2023 by sex, geographical region, and human development index (HDI), in China

Characteristics	Relative change in number of patients [% (95% CI)]	Relative change in number of YLDs [% (95% CI)]	AAPC in ASR of prevalence (95% CI)	AAPC in ASR of YLDs (95% CI)
All	163.36 (142.59 – 185.22)	163.36 (157.32 – 185.22)	3.36 (3.01 – 3.65)	4.68 (4.44 – 4.98)
Sex				
Male	155.53 (135.58 – 176.31)	155.52 (149.79 – 161.31)	3.33 (3.00 – 3.62)	4.53 (4.31 – 4.81)
Female	172.81 (151.04 – 195.97)	172.82 (166.40 – 179.27)	3.17 (2.70 – 3.57)	4.85 (4.59 – 5.18)
Region				
North China	147.31 (127.80 – 167.43)	147.31 (142.42 – 152.24)	2.84 (2.64 – 3.03)	4.21 (4.03 – 4.40)
Northeast China	103.64 (87.77 – 120.24)	103.62 (99.46 – 107.82)	2.65 (2.46 – 2.82)	4.52 (4.36 – 4.72)
East China	185.02 (162.52 – 208.90)	185.03 (178.84 – 191.26)	3.72 (3.43 – 4.02)	4.88 (4.71 – 5.14)
South Central China	159.35 (138.82 – 181.01)	159.34 (152.56 – 166.17)	2.95 (2.71 – 3.20)	4.25 (3.88 – 4.70)
Southwest China	194.53 (171.22 – 218.69)	194.56 (187.25 – 201.86)	4.12 (3.82 – 4.44)	5.46 (5.29 – 5.74)
Northwest China	168.69 (147.63 – 191.54)	168.68 (161.70 – 175.69)	3.09 (2.73 – 3.45)	4.68 (4.44 – 4.96)
HDI quintile (highest to lowest)				
1	186.91 (164.19 – 209.84)	186.88 (180.97 – 192.83)	3.09 (2.60 – 3.52)	4.30 (3.95 – 4.75)
2	189.44 (167.12 – 214.36)	189.45 (182.78 – 196.13)	3.79 (3.37 – 4.18)	5.44 (5.22 – 5.70)
3	138.85 (120.39 – 159.18)	138.85 (133.08 – 144.67)	2.84 (2.58 – 3.08)	4.36 (4.10 – 4.67)
4	148.02 (128.10 – 169.01)	148.04 (142.46 – 153.65)	3.18 (2.96 – 3.41)	4.66 (4.46 – 4.97)
5	164.89 (143.47 – 185.95)	164.94 (156.46 – 173.49)	3.21 (2.91 – 3.53)	4.83 (4.55 – 5.18)

Table S4 The number of cases, age-standardized rate (ASR) of prevalence, years lived with disability (YLDs), and ASR of YLDs for diabetes in 2023, as well as annual average percentage change (AAPC) in the ASR of prevalence and YLDs during 2005 – 2023, in Chinese 31 provinces (autonomous regions and municipalities)

**Provinces**	**Patients [thousands (95% CI)]**	**ASR of prevalence [% (95% CI)]**	**AAPC in ASR of prevalence (95% CI)**	**YLDs [thousands (95% CI)]**	**ASR of YLDs [per 1000 population (95% CI)]**	**AAPC in ASR of YLDs (95% CI)**
North China						
Beijing	8116.04 (7458.26 – 8818.11)	28.45 (26.15 – 30.91)	3.37 (2.99 – 3.72)	1131.76 (1115.07 – 1148.35)	39.67 (39.03 – 40.32)	4.57 (4.21 – 5.00)
Tianjin	4617.79 (4290.77 – 4964.73)	25.78 (23.95 – 27.71)	2.38 (2.08 – 2.66)	643.85 (634.06 – 653.69)	35.94 (35.33 – 36.56)	3.51 (3.27 – 3.79)
Hebei	12897.33 (11859.71 – 13907.46)	14.55 (13.38 – 15.70)	2.30 (2.07 – 2.52)	1798.58 (1758.49 – 1839.19)	20.29 (19.80 – 20.79)	3.54 (3.23 – 3.91)
Shanxi	6502.33 (5987.36 – 7038.06)	15.31 (14.10 – 16.56)	2.60 (2.23 – 2.97)	906.63 (886.98 – 926.52)	21.34 (20.83 – 21.85)	4.53 (4.20 – 4.76)
Inner Mongolia	3926.44 (3618.42 – 4264.20)	12.58 (11.59 – 13.66)	3.36 (3.09 – 3.62)	547.47 (534.23 – 560.69)	17.54 (17.07 – 18.01)	5.14 (4.88 – 5.44)
Northeast China						
Liaoning	9281.83 (8588.45 – 10013.55)	15.52 (14.35 – 16.74)	2.40 (2.17 – 2.64)	1294.07 (1267.52 – 1321.16)	21.64 (21.13 – 22.16)	3.98 (3.73 – 4.33)
Jilin	5177.39 (4788.75 – 5600.77)	16.03 (14.84 – 17.36)	2.80 (2.61 – 2.99)	721.98 (707.48 – 736.54)	22.36 (21.85 – 22.87)	4.60 (4.43 – 4.83)
Heilongjiang	6656.19 (6092.81 – 7223.25)	15.43 (14.12 – 16.75)	2.74 (2.56 – 2.92)	928.15 (909.07 – 947.22)	21.51 (21.00 – 22.02)	4.76 (4.60 – 4.96)
East China						
Shanghai	7406.93 (6803.72 – 7959.06)	22.04 (20.25 – 23.69)	3.16 (2.76 – 3.70)	1032.79 (1015.40 – 1050.53)	30.73 (30.16 – 31.32)	4.15 (3.78 – 4.66)
Jiangsu	17990.07 (16523.65 – 19637.23)	16.35 (15.01 – 17.84)	4.08 (3.84 – 4.29)	2508.08 (2456.75 – 2560.25)	22.79 (22.28 – 23.32)	5.21 (5.02 – 5.43)
Zhejiang	14854.39 (13669.50 – 15980.26)	17.72 (16.31 – 19.07)	3.46 (3.15 – 3.83)	2070.98 (2029.62 – 2112.51)	24.71 (24.18 – 25.25)	4.61 (4.37 – 4.96)
Anhui	11350.23 (10455.77 – 12423.82)	15.42 (14.21 – 16.88)	3.84 (3.54 – 4.12)	1582.59 (1548.41 – 1617.10)	21.50 (21.00 – 22.02)	5.30 (5.06 – 5.66)
Fujian	8034.35 (7408.58 – 8674.81)	16.64 (15.35 – 17.97)	4.14 (3.75 – 4.54)	1120.39 (1096.78 – 1144.22)	23.21 (22.69 – 23.73)	5.41 (5.10 – 5.88)
Jiangxi	5088.06 (4665.03 – 5545.88)	10.19 (9.35 – 11.11)	3.08 (2.39 – 3.74)	709.46 (689.53 – 729.64)	14.21 (13.79 – 14.64)	4.94 (4.51 – 5.72)
Shandong	13745.28 (12748.96 – 14821.03)	10.93 (10.14 – 11.79)	3.09 (2.81 – 3.36)	1916.77 (1867.11 – 1966.06)	15.25 (14.81 – 15.68)	4.13 (3.90 – 4.41)
South Central						
Henan	15992.04 (14761.18 – 17424.91)	14.33 (13.23 – 15.62)	2.79 (2.44 – 3.13)	2229.89 (2178.81 – 2281.55)	19.98 (19.49 – 20.48)	3.94 (3.66 – 4.25)
Hubei	8765.97 (8081.10 – 9567.60)	12.03 (11.08 – 13.12)	3.67 (3.12 – 4.19)	1222.28 (1191.95 – 1252.63)	16.78 (16.32 – 17.24)	5.23 (4.94 – 5.65)
Hunan	8077.13 (7405.19 – 8802.75)	10.09 (9.24 – 10.99)	3.47 (3.20 – 3.73)	1126.19 (1095.76 – 1157.32)	14.07 (13.65 – 14.49)	4.62 (4.34 – 5.01)
Guangdong	16022.12 (14754.05 – 17172.41)	11.68 (10.75 – 12.52)	2.65 (2.40 – 2.91)	2234.17 (2172.52 – 2295.61)	16.28 (15.83 – 16.74)	4.07 (3.70 – 4.56)

Provinces	Patients [thousands (95% CI)]	ASR of prevalence [% (95% CI)]	AAPC in ASR of prevalence (95% CI)	YLDs [thousands (95% CI)]	ASR of YLDs [per 1000 population (95% CI)]	AAPC in ASR of YLDs (95% CI)
Guangxi	3950.44 (3615.50 – 4251.34)	7.23 (6.61 – 7.78)	2.51 (2.18 – 2.85)	550.75 (532.16 – 569.64)	10.07 (9.71 – 10.44)	3.55 (3.27 – 3.86)
Hainan	1173.53 (1091.40 – 1270.02)	10.34 (9.61 – 11.19)	2.85 (2.52 – 3.16)	163.59 (158.97 – 168.41)	14.41 (13.99 – 14.85)	4.19 (3.91 – 4.50)
Southwest China						
Chongqing	6457.31 (5971.38 – 7022.66)	15.51 (14.34 – 16.87)	4.41 (3.97 – 4.81)	900.28 (881.01 – 919.34)	21.63 (21.12 – 22.14)	5.59 (5.29 – 5.96)
Sichuan	15146.25 (13924.63 – 16394.00)	14.07 (12.92 – 15.22)	4.13 (3.89 – 4.44)	2111.88 (2064.52 – 2159.11)	19.62 (19.13 – 20.10)	5.50 (5.33 – 5.78)
Guizhou	3091.94 (2832.38 – 3341.77)	7.57 (6.94 – 8.19)	3.85 (3.42 – 4.28)	431.19 (416.58 – 445.99)	10.56 (10.18 – 10.93)	5.40 (5.01 – 5.92)
Yunnan	4788.54 (4422.44 – 5143.88)	9.13 (8.43 – 9.81)	3.76 (3.55 – 3.99)	667.77 (647.57 – 688.01)	12.73 (12.32 – 13.13)	5.32 (5.12 – 5.61)
Xizang	135.27 (124.60 – 146.78)	3.93 (3.62 – 4.26)	3.80 (3.10 – 4.62)	18.86 (17.91 – 19.86)	5.48 (5.21 – 5.75)	5.00 (4.35 – 5.92)
Northwest China						
Shaanxi	5344.51 (4908.78 – 5817.96)	11.11 (10.21 – 12.10)	3.68 (3.36 – 3.98)	745.13 (725.58 – 764.74)	15.49 (15.06 – 15.94)	5.06 (4.86 – 5.33)
Gansu	2412.85 (2219.51 – 2627.84)	8.19 (7.53 – 8.92)	3.30 (3.11 – 3.49)	336.43 (325.87 – 346.97)	11.42 (11.03 – 11.80)	4.94 (4.76 – 5.10)
Qinghai	620.55 (573.28 – 670.57)	9.70 (8.96 – 10.49)	2.80 (2.46 – 3.14)	86.51 (83.93 – 89.08)	13.53 (13.11 – 13.94)	4.27 (3.94 – 4.64)
Ningxia	871.29 (799.95 – 950.22)	10.89 (10.00 – 11.88)	2.88 (2.23 – 3.45)	121.50 (118.16 – 124.85)	15.19 (14.76 – 15.62)	4.50 (4.06 – 5.08)
Xinjiang	4540.28 (4207.03 – 4895.24)	16.56 (15.34 – 17.86)	2.27 (1.66 – 2.83)	633.13 (619.18 – 647.17)	23.09 (22.58 – 23.61)	3.54 (3.08 – 4.18)

Table S5 Age-standardized rate (ASR) of prevalence for diabetes among adults aged 18 and above

Study	Standard populations	Year	ASR of prevalence [% (95% CI)]
This study	2020 national population census	2018	16.40 (15.22 – 17.70)
2018 CCDRFS	2020 national population census	2018	14.83 (12.03 – 15.74)
GBD 2021	2020 national population census	2018	10.00 (8.85 – 11.29)

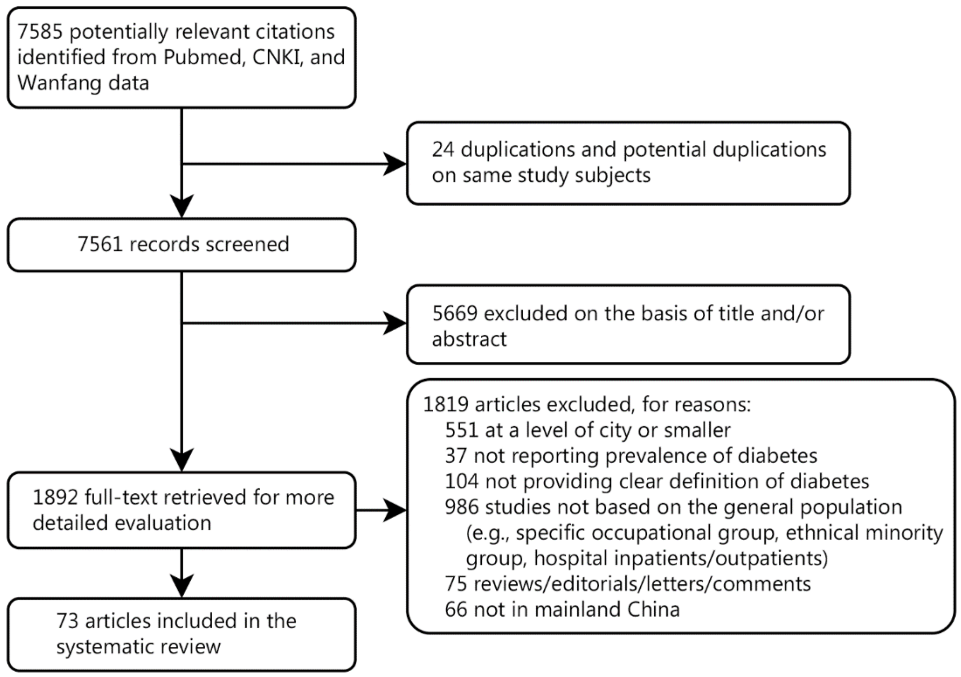

Fig. S1 Systematic review flow diagram for selection of studies

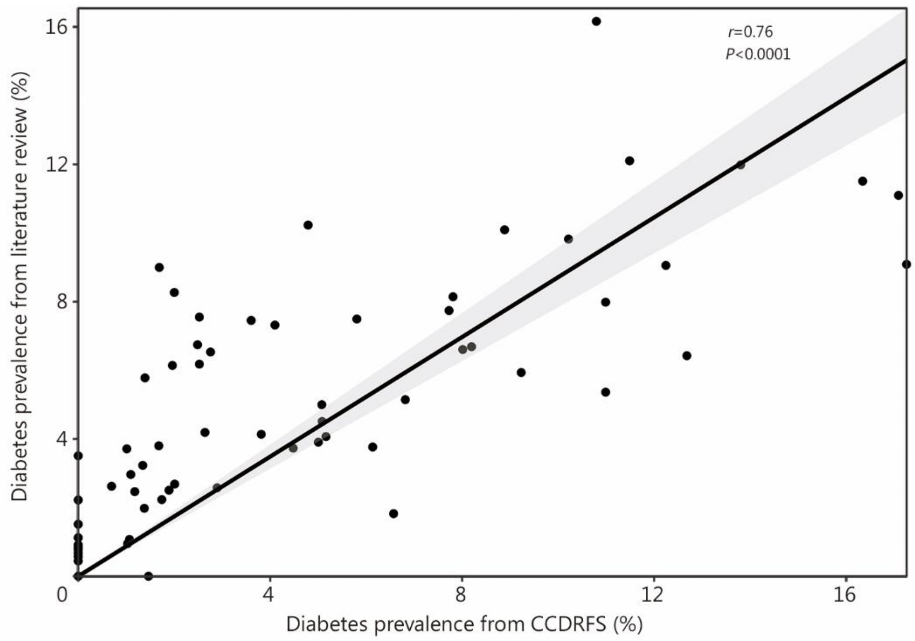

Fig. S2 Comparison of the prevalence of diabetes from a literature review and the China Chronic Disease Control and Risk Factor Surveillance (CCDRFS) (after Crosswalk process). The data points are matched by location, year, and age group

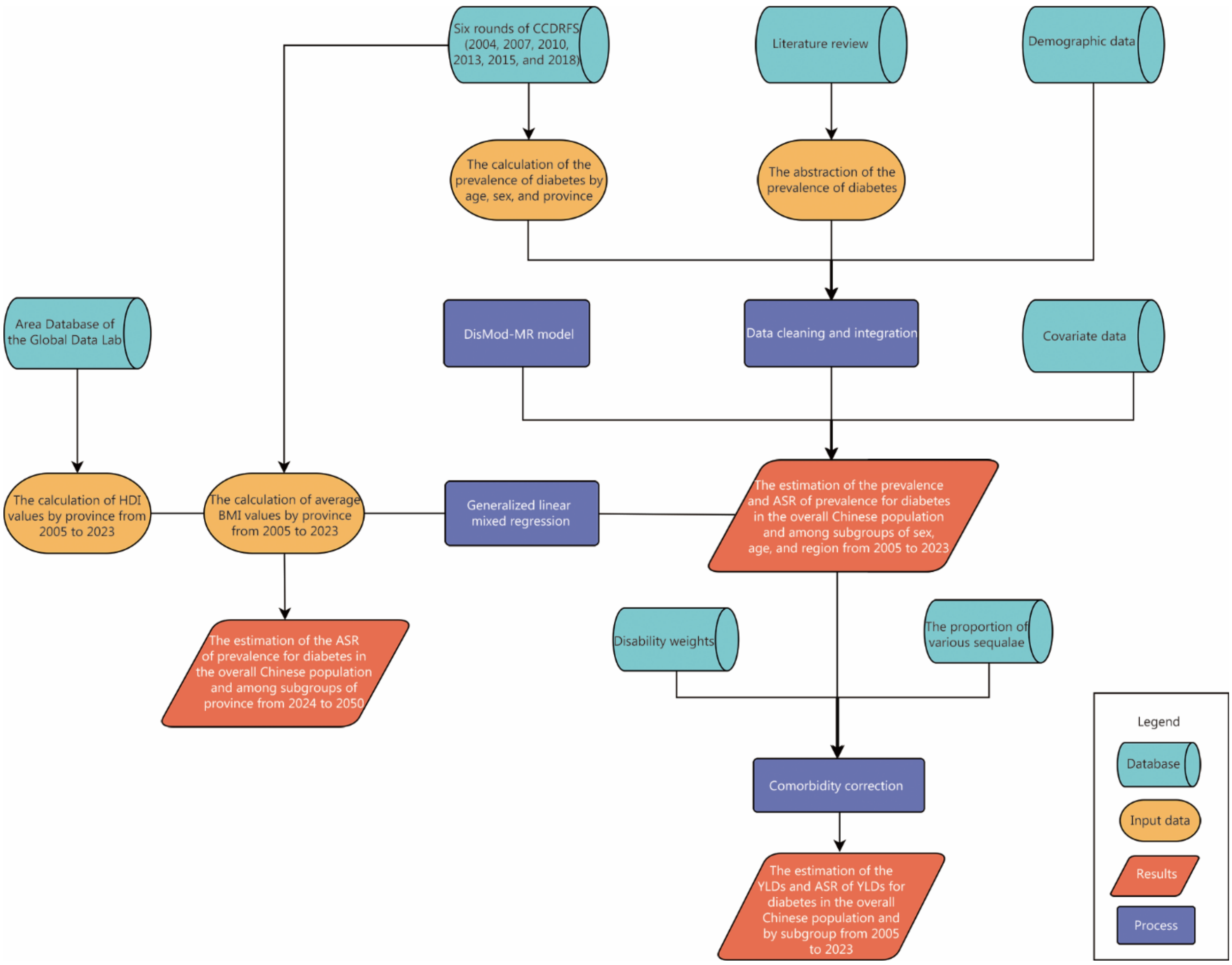

Fig. S3 The diagram of the estimation process of prevalence and YLDs of diabetes. CCDRFS China Chronic Disease and Risk Factors Surveillance, ASR age-standardized rate, ASPR age-standardized prevalence rate, YLDs years lived with disability, HDI human development index

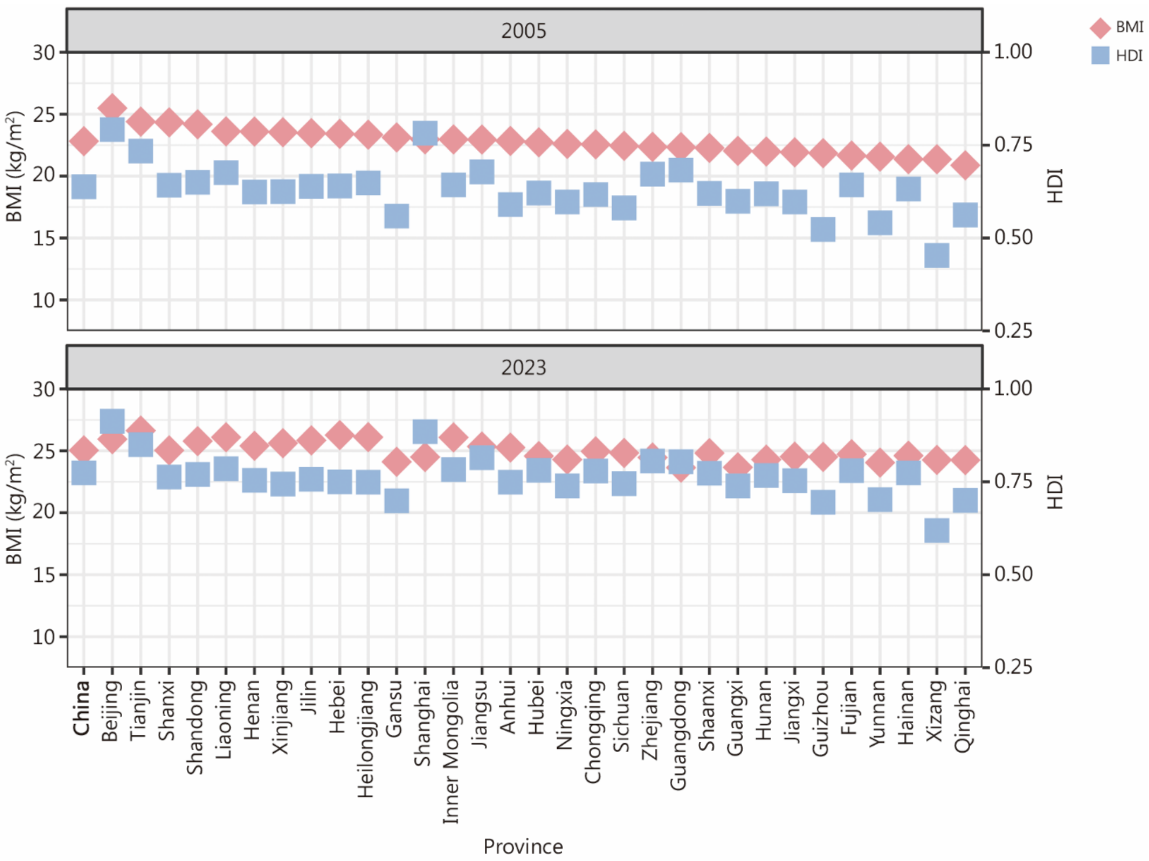

Fig. S4 The body mass index (BMI) and human development index (HDI) in China and by province in 2005 and 2023

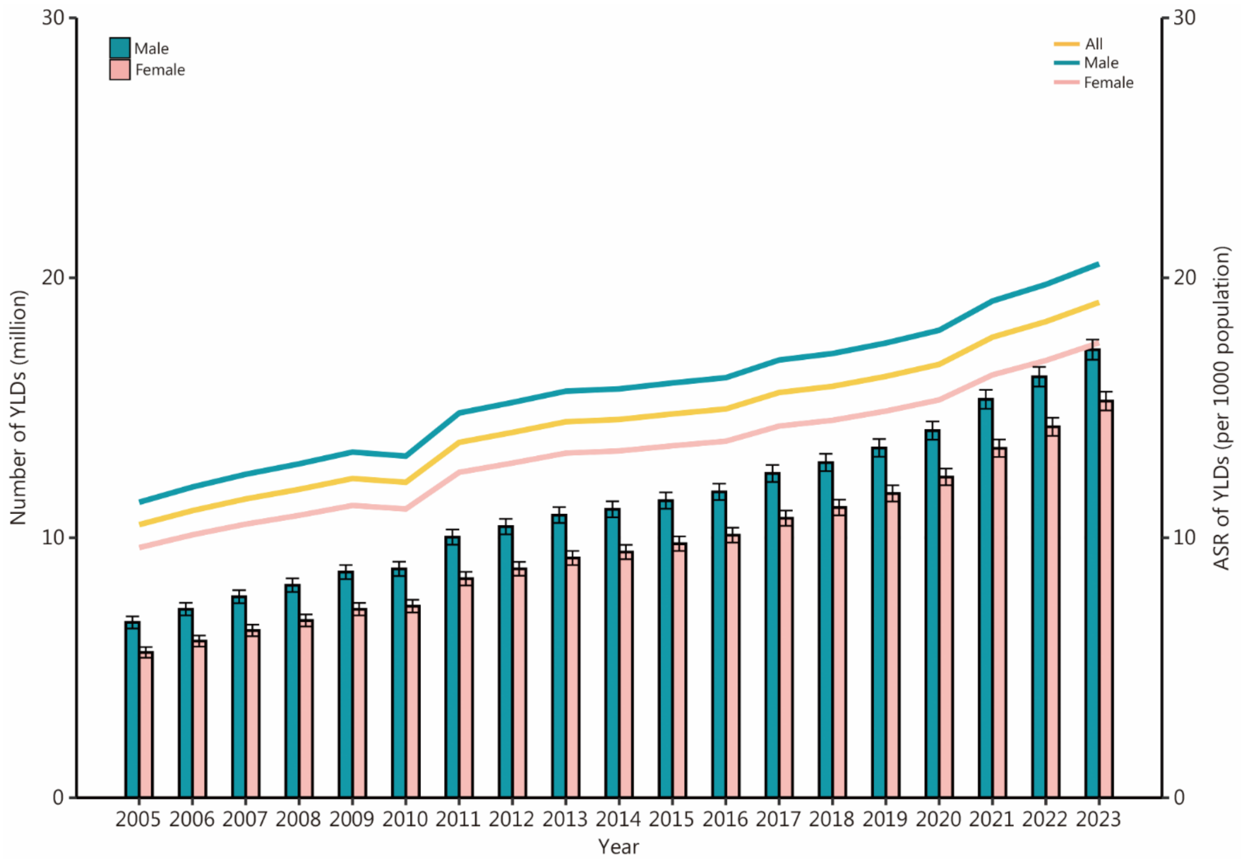

Fig. S5 The number of years lived with disability (YLDs) and age-standardized rate (ASR) of YLDs for diabetes by sex in China from 2005 to 2023

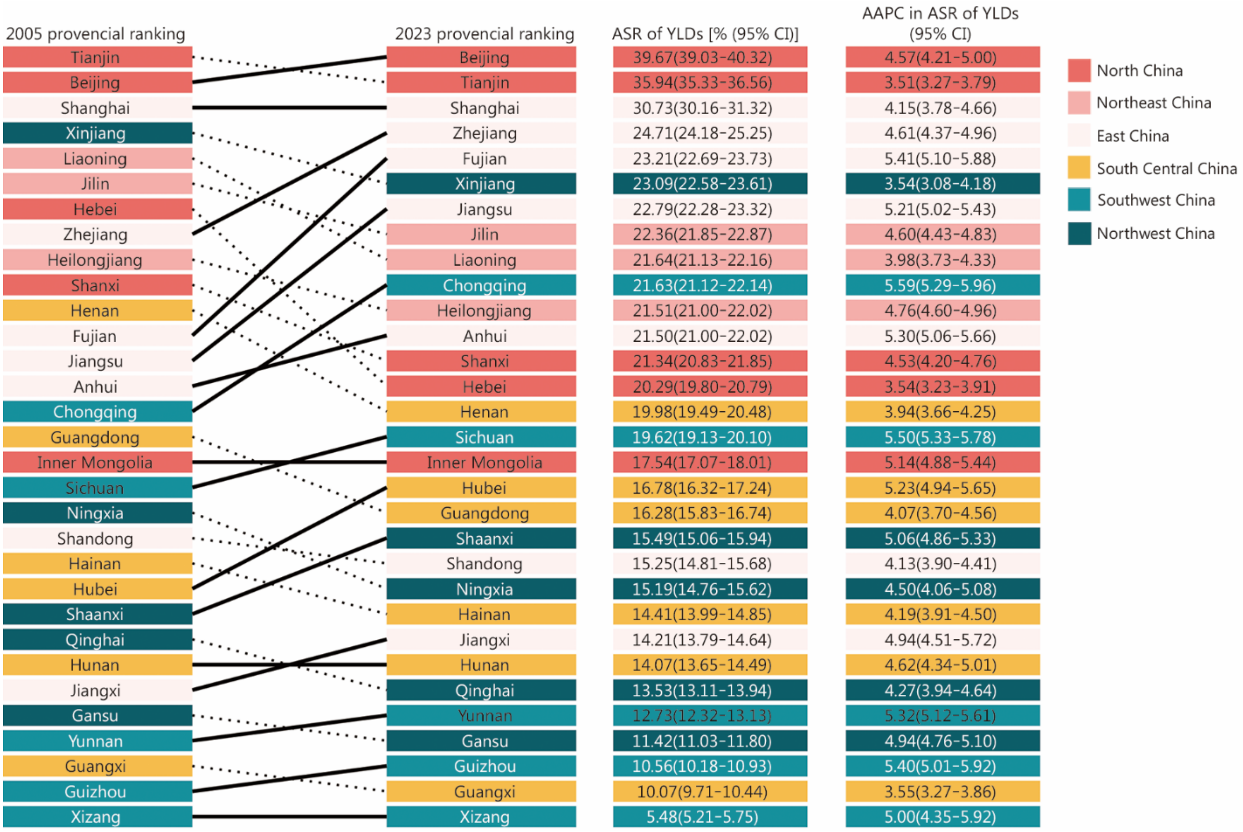

Fig. S6 Age-standardized rate (ASR) of years lived with disability (YLDs) for diabetes in China at the provincial level in 2023, and annual average percentage change (AAPC) in ASR of YLDs from 2005 to 2023

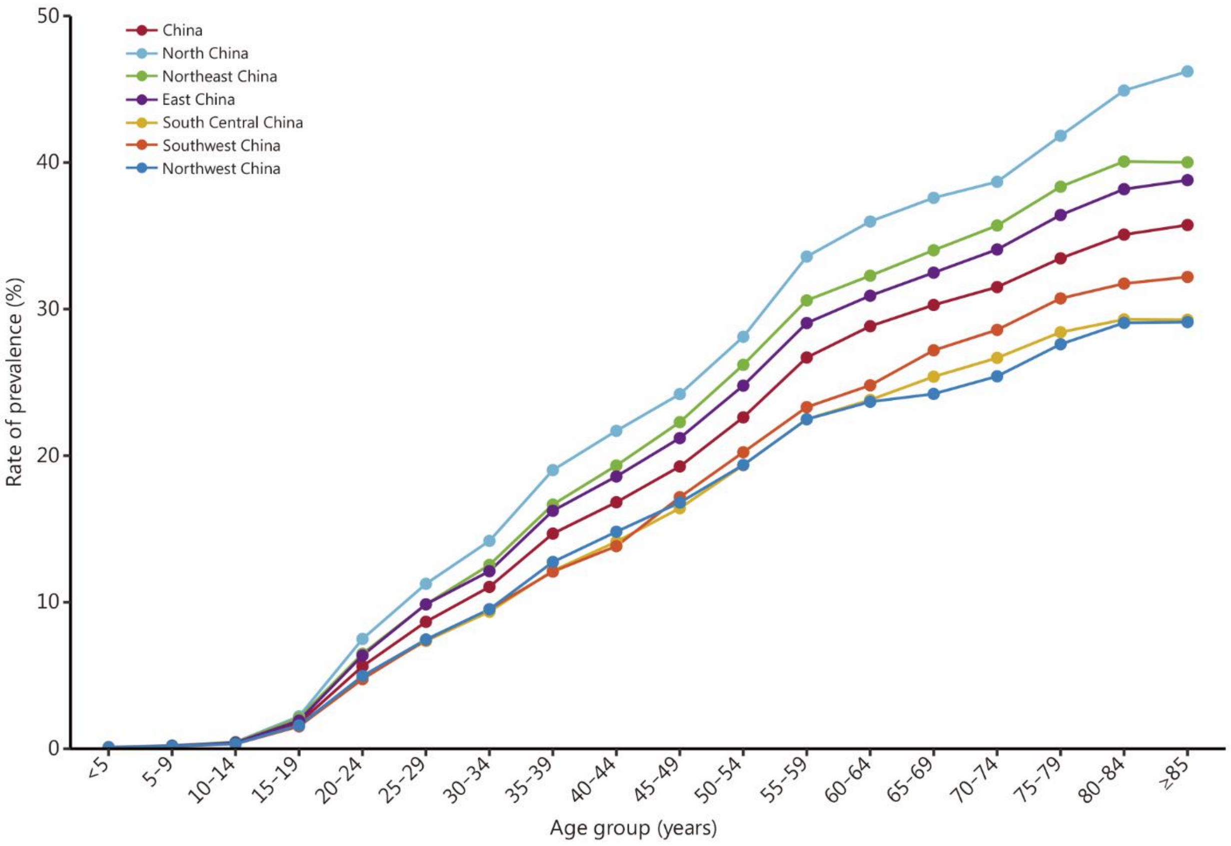

Fig. S7 The prevalence of diabetes by 5-year age groups in China and Chinese six regions, 2023

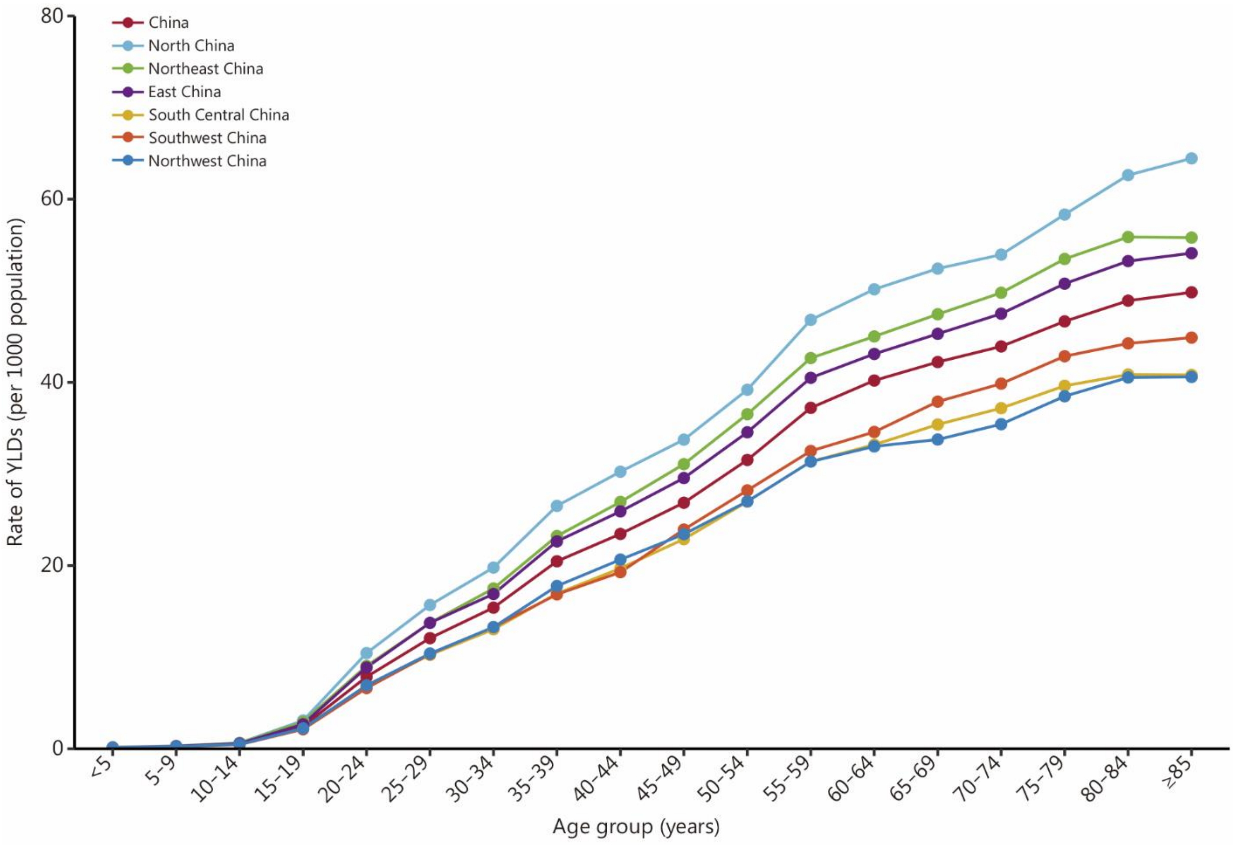

Fig. S8 The rate of years lived with disability (YLDs) by 5-year age groups in China and Chinese six regions, 2023

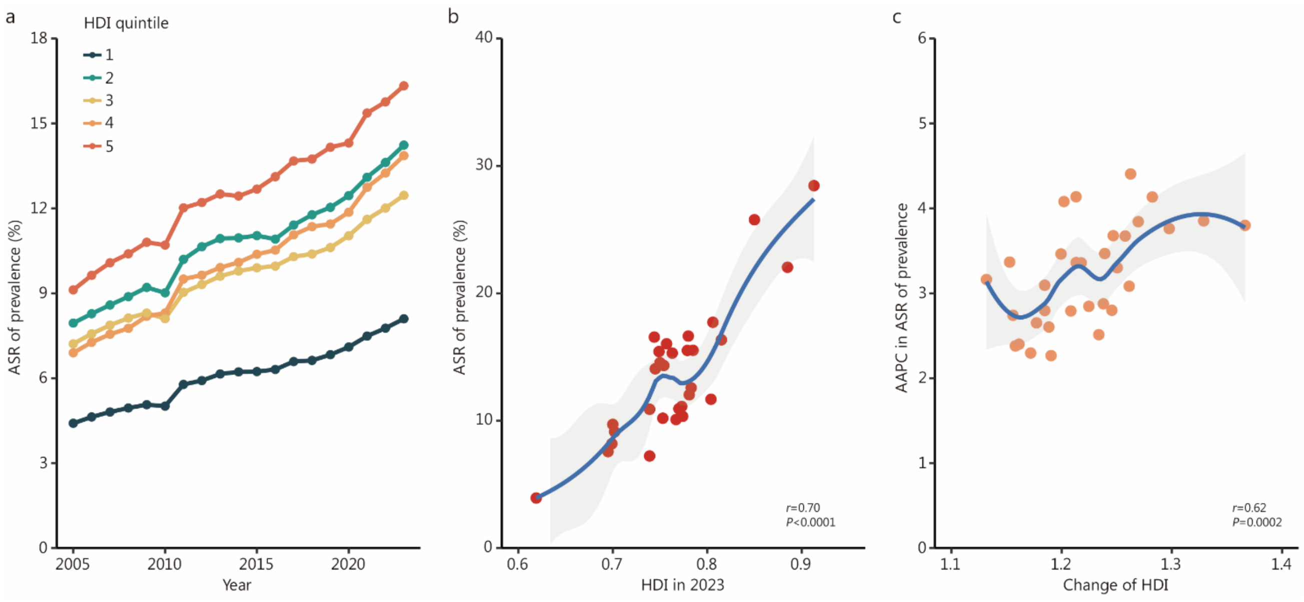

Fig. S9 Age-standardized rate (ASR) of prevalence for diabetes by quintile of human development index (HDI) during 2005 – 2023 (**a**), the correlation between HDI and ASR of prevalence in 2023 (**b**), and the correlation between the change of HDI and annual average percentage change (AAPC) in ASR of prevalence during 2005 – 2023 (**c**). The blue lines separately indicate the expected ASR of prevalence based solely on the HDI, and the anticipated AAPC in the ASR of prevalence, considering changes in the HDI alone

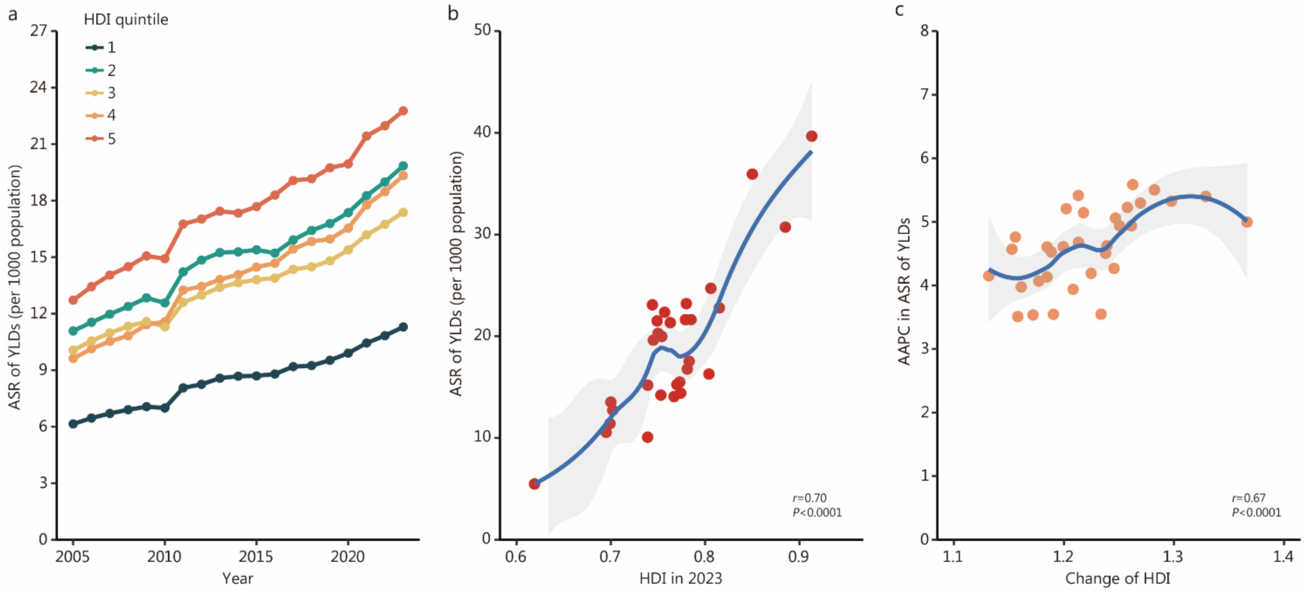

Fig. S10 Age-standardized rate (ASR) of YLDs for diabetes by quintile of human development index (HDI) during 2005 – 2023 (**a**), the correlation between HDI and ASR of YLDs in 2023 (**b**), and the correlation between the change of HDI and annual average percentage change (AAPC) in ASR of YLDs during 2005 – 2023 (**c**). The blue lines separately indicate the expected ASR of YLD based solely on the HDI, and the anticipated AAPC in the ASR of YLD, considering changes in the HDI alone

References

1. National Center for Chronic and Noncommunicable Disease Control and Prevention, Chinese Center for Disease Control and Prevention. [Report on Chronic Disease Risk Factor Surveillance in China, 2004]. Beijing: Peking Union Medical College Press; 2009. Chinese.
2. Chinese Center for Disease Control and Prevention. [Report on Chronic Disease Risk Factor Surveillance in China, 2007]. Beijing: People’s Medical Publishing House; 2010. Chinese.
3. Chinese Center for Disease Control and Prevention. [Report on Chronic Disease Risk Factor Surveillance in China, 2010]. Beijing: Publication House of Military Medical Sciences; 2012. Chinese.
4. Chinese Center for Disease Control and Prevention. [Report on Chronic Disease Risk Factor Surveillance in China, 2013]. Beijing: Publication House of Military Medical Sciences; 2016. Chinese.
5. National Health and Family Planning Commission of the People’s Republic of China. [Report on the nutrition and chronic disease status of Chinese residents, 2015]. Beijing: People’s Medical Publishing House; 2015. Chinese
6. Liu S, Wu X, Lopez AD, Wang L, Cai Y, Page A, et al. An integrated national mortality surveillance system for death registration and mortality surveillance, China. B World Health Organ. 2016;94(1):46-57.
7. Wang L, Gao P, Zhang M, Huang Z, Zhang D, Deng Q, et al. Prevalence and ethnic pattern of diabetes and prediabetes in China in 2013. JAMA. 2017;317(24):2515-23.
8. Zhang M, Wang L, Wu J, Huang Z, Zhao Z, Zhang X, et al. Data resource profile: China Chronic Disease and Risk Factor Surveillance (CCDRFS). Int J Epidemiol. 2022;51(2):e1-8.
9. Li Y, Zhang M, Jiang Y, Wu F. Co-variations and clustering of chronic disease behavioral risk factors in China: China Chronic Disease and Risk Factor Surveillance, 2007. PLoS One. 2012;7(3):e33881.
10. Liu S, Zhang M, Yang L, Li Y, Wang L, Huang Z, et al. Prevalence and patterns of tobacco smoking among Chinese adult men and women: findings of the 2010 national smoking survey. J Epidemiol Commun H. 2017;71(2):154-61.
11. Zhang M, Deng Q, Wang L, Huang Z, Zhou M, Li Y, et al. Prevalence of dyslipidemia and achievement of low-density lipoprotein cholesterol targets in Chinese adults: a nationally representative survey of 163,641 adults. Int J Cardiol. 2018;260:196-203.
12. Xu Y. Prevalence and control of diabetes in Chinese adults. JAMA. 2013;310(9):948.
13. Stroup DF, Berlin JA, Morton SC, Olkin I, Williamson GD, Rennie D, et al. Meta-analysis of observational studies in epidemiology: a proposal for reporting. Meta-analysis of Observational Studies in Epidemiology (MOOSE) group. JAMA. 2000;283(15):2008-12.

14. GBD 2021 Diseases and Injuries Collaborators. Global incidence, prevalence, years lived with disability (YLDs), disability-adjusted life-years (DALYs), and healthy life expectancy (HALE) for 371 diseases and injuries in 204 countries and territories and 811 subnational locations, 1990 – 2021: a systematic analysis for the Global Burden of Disease Study 2021. Lancet. 2024;403(10440):2133-61.
15. GBD 2019 Diseases and Injuries Collaborators. Global burden of 369 diseases and injuries in 204 countries and territories, 1990 – 2019: a systematic analysis for the Global Burden of Disease Study 2019. Lancet. 2020;396(10258):1204-22.
16. Liu X, Wang F, Yu C, Zhou M, Yu Y, Qi J, et al. Eliciting national and subnational sets of disability weights in mainland China: findings from the Chinese disability weight measurement study. Lancet Reg Health West Pac. 2022;26:100520.
17. Song P, Yu J, Chan KY, Theodoratou E, Rudan I. Prevalence, risk factors and burden of diabetic retinopathy in China: a systematic review and meta-analysis. J Glob Health. 2018;8(1):010803.
18. Guo L, Ye L, Yuan J. [Prevalence and influencing factors of diabetes for the 45 years and older population in China]. Mod Prevent Med. 2019;46(16):2890-4. Chinese.
19. Chi X, Yu D, Ju L, Zhang J, Zhao L. [Prevalence of diabetes and change among 7 to 17 years old children and adolescents in China in 2002-2012]. J Hygiene Res. 2018;47(5):705-9. Chinese.
20. Liu S, Wang J. Estimation of the prevalence of diabetes mellitus and the rate of impaired fasting glucose for Chinese adults. Chin J Prev Control Chronic Dis. 2010;18(4):331-4. Chinese.
21. Bragg F, Holmes MV, Iona A, Guo Y, Du H, Chen Y, et al. Association between diabetes and cause-specific mortality in rural and urban areas of China. JAMA. 2017;317(3):280.
22. Liu C, Yang C, Zhao Y, Ma Z, Bi J, Liu Y, et al. Associations between long-term exposure to ambient particulate air pollution and type 2 diabetes prevalence, blood glucose and glycosylated hemoglobin levels in China. Environ Int. 2016;92-93:416-21.
23. Wu J, Cheng X, Qiu L, Xu T, Zhu G, Han J, et al. Prevalence and clustering of major cardiovascular risk factors in China. Medicine. 2016;95(10):e2712.
24. Bu S, Ruan D, Yang Z, Xing X, Zhao W, Wang N, et al. Sex-specific prevalence of diabetes and cardiovascular risk factors in the middle-aged population of China: a subgroup analysis of the 2007 – 2008 China national diabetes and metabolic disorders study. PLoS One. 2015;10(9):e139039.
25. Yang W, Lu J, Weng J, Jia W, Ji L, Xiao J, et al. Prevalence of diabetes among men and women in China. N Engl J Med. 2010;362(12):1090-101.
26. Wang T, Xu Y, Xu M, Wang W, Bi Y, Lu J, et al. Awareness, treatment and control of cardiometabolic disorders

in Chinese adults with diabetes: a national representative population study. Cardiovasc Diabetol. 2015;14(1):28.
27. Mezuk B, Chen Y, Yu C, Guo Y, Bian Z, Collins R, et al. Depression, anxiety, and prevalent diabetes in the Chinese population: findings from the China Kadoorie Biobank of 0.5 million people. J Psychosom Res. 2013;75(6):511-7.
28. Attard SM, Herring AH, Mayer-Davis EJ, Popkin BM, Meigs JB, Gordon-Larsen P. Multilevel examination of diabetes in modernising China: what elements of urbanisation are most associated with diabetes?. Diabetologia. 2012;55(12):3182-92.
29. Li Y. Epidemiological study on prevalence and risk factors of thyroid disorders and diabetes among adults in China. Shenyang: China Medical University; 2021.
30. Wang L, Peng W, Zhao Z, Zhang M, Shi Z, Song Z, et al. Prevalence and treatment of diabetes in China, 2013-2018. JAMA. 2021;326(24):2498-506.
31. Bai A, Tao J, Tao L, Liu J. Prevalence and risk factors of diabetes among adults aged 45 years or older in China: a national cross-sectional study. Endocrinol Diabetes Metab. 2021;4(3):e265.
32. Jin C, Lai Y, Li Y, Teng D, Yang W, Teng W, et al. Changes in the prevalence of diabetes and control of risk factors for diabetes among Chinese adults from 2007 to 2017: an analysis of repeated national cross-sectional surveys. J Diabetes. 2024;16(2):e13492.
33. Fang K, Ma A, Li H, Dong J, Xie J, Xie C, et al. [Prevalence and risk factors of diabetes mellitus and impaired fasting glucose among residents aged 40 – 79 years in Beijing]. Chin Gener Pract. 2019;22(9):1014-20. Chinese.
34. Wang Z, Wu Y, Wu J, Wang M, Wang X, Wang J, et al. Trends in prevalence and incidence of type 2 diabetes among adults in Beijing, China, from 2008 to 2017. Diabet Med. 2021;38(9):e14487.
35. Cao B, Mi J, Gong C, Cheng H, Yan C, Hou D, et al. [The prevalence of diabetes in children and adolescents of Beijing]. Chin J Epidemiol. 2007;28(7):631-4. Chinese.
36. Zhan Y, Yu J, Hu D, Sun Y, Fu Y, Zhang L, et al. [A cross-sectional study: the prevalence of impaired fasting glucose and diabetes mellitus among residents, Beijing]. Chin Prev Med. 2010;11(12):1218-21. Chinese.
37. Wei J, Liu F, Zhou L, Chen Y, Tong X, Liu Y, et al. [Beijing municipal epidemiological investigation on hazards of abnormal glucose tolerance and diabetes as well as their Chinese syndromes]. Beijing J Tradit Chin Med. 2010;29(10):731-7. Chinese.
38. Zhang P, Jiao S, Zhou Y, Li G, Shi Y, Li H, et al. [Studies on prevalence and control of several common chronic diseases among Beijing adults in 2005]. Chin J Epidemiol. 2007;28(7):625-30. Chinese.
39. Liu M, Wang J, He Y, Jiang B, Wu L, Wang Y, et al. Awareness, treatment and control of type 2 diabetes among Chinese elderly and its changing trend for past decade. Bmc Public Health. 2016;16(1):278.

40. Zhi X, Wang J. [A study of prevalence and its risk factors of type 2 diabetes in Tianjin]. Chin J Diabetes. 2009;17(04):275-7. Chinese.

41. Chang G, Tian Y, Wang D, Li W, Wang G. [Analysis on current status and influential factors of diabetes among residents from different districts in Tianjin]. Chin J Prevent Control Chronic Dis. 2011;19(2):144-7. Chinese.

42. Qian D, Wang Y, Wang G, Qi X. [Prevalence rate of type 2 diabetes in community residents and its related factors:a cross-sectional study]. Chin J Pub Health. 2009;25(12):1441-2. Chinese.

43. Li W, Jiang G, Wu T, Wang Z. [The Prevalence of diabetes mellitus and its effect factors among rural and urban residents of Tianjin]. J Occup Health Damage. 2009;25(8):828-30. Chinese.

44. You Y, Lu C, Pan L, Ma Y, Gao Q, Li N. [Analysis of the prevalence and influencing factors of diabetes mellitus among adults in Liaoning province]. Chin J Dis Control Prev. 2018;22(1):19-22. Chinese.

45. You Y, Li N, Wu M, Pan L, Ma Y, Gao Q, et al. [Prevalence and influencing factors of main chronic diseases among urban residents in Liaoning province]. J Prev Med. 2018;30(1):35-40. Chinese.

46. Yang BY, Qian ZM, Li S, Chen G, Bloom MS, Elliott M, et al. Ambient air pollution in relation to diabetes and glucose-homoeostasis markers in China: a cross-sectional study with findings from the 33 communities Chinese health study. Lancet Planet Health. 2018;2(2):e64-73.

47. Pan L, Lu C, Wu M, You Y, Guo J, Ma Y, et al. Analysis on the diabetes prevalence and its influencing factors in population aged 35 – 75 years in Liaoning province. Chin J Dis Control Prev. 2020;24(6):670-5. Chinese.

48. Su Y, Ma Y, Rao W, Yang G, Wang S, Fu Y, et al. Association between body mass index and diabetes in Northeastern China: based on dose-response analyses using restricted cubic spline functions. Asia-Pac J Public He. 2016;28(6):486-97.

49. Yu J, Ma Y, Yang S, Pang K, Yu Y, Tao Y, et al. [Risk factors for Cardiovascular disease and their clustering among adults in Jilin]. Int J Env Res Pub He. 2016;13(1):70. Chinese.

50. Xing L, Tian Y, Jing L, Lin M, Du Z, Sun Q, et al. Status and disparities of diabetes among urban and rural residents aged 40 years and older: insight from a population-based study in northeast China, 2017 – 2019. J Epidemiol Community Health. 2021;75(8):800-8.

51. Zhao S, Hou Y, Rao H, Zhao M, Guo J, Liu X, et al. [Cross-sectional study on diabetes mellitus among urban and rural residents in Shanxi province]. Chin Prev Med. 2009;10(1):5-9. Chinese.

52. He L, La Y, Yan Y, Wang Y, Cao X, Cai Y, et al. [The prevalence and burden of four major chronic diseases in the Shanxi province of Northern China]. Front Public Health. 2022;10:985192. Chinese.

53. Yang J, Lu Y, Zhu J, Wu S, Li J, Wang M, et al. [An epidemiological survey on diabetes mellitus in Anhui province]. J Fuyang Teachers College (Natural Science Edition). 2017;34(1):56-60. Chinese.

54. Ding X, Zhuo-Zhi S, Mao D, Zhang C, Lv X, Jiao Y. [Analysis on diabetes morbidity and its risk factors among adults in Chongqing city]. Chin J Prev Control Chronic Dis. 2016;24(1):1-4. Chinese.
55. Wang L, Li Q, Fan J, Zhang S, Cheng D, Zhong L, et al. [Investigation and analysis of risk factors associated with type 2 diabetes mellitus in the Chongqing community in 2008]. Chongqing Med. 2009;38(18):2277-80. Chinese.
56. Gao L, Zhou G, Feng S, Feng H, Han B, Wang C. [Epidemiological investigation of diabetes among residents in Henan]. J Zhengzhou Univ (Med Sci). 2014;49(1):123-6. Chinese.
57. Tian R, Zhao Z, Yuan H, Wang Y, Qin G, Yan Y, et al. [Epidemiologic survey and correlation factors analysis of diabetes mellitus and metabolic syndrome in Henan province]. J Chine Practi Diag Therapy. 2009;23(11):1052-5. Chinese.
58. Li H, Qi M, Zhang H, An W, Fan L, Feng S, et al. [The prevalence, awareness, treatment and control of diabetes in population aged above 60 years old in Henan province]. Chin J Health Educ. 2022;38(12):1066-71. Chinese.
59. Zhu S, Zhang H, Yu G, Niu J. [The epidemic characteristics of overweight and obesity and relation with the prevalence of diabetes in Gansu province]. Chin J Pub Health Eng. 2013;12(2):98-100. Chinese.
60. Hao L. [Epidemiology of Diabetes in adults in northwest China’s Gansu province]. Lanzhou: Lanzhou University; 2015. Chinese.
61. Chen X, Guo X, Tang J, Ma J, Lu Z, Zhang J, et al. [Analysis of epidemiological feature and risk factors of diabetes mellitus and impaired glucose regulation in residents of Shandong province, China]. Chin Prev Med. 2012;13(9):683-8. Chinese.
62. Liang K, Sun Y, Li W, Zhang X, Li C, Yang W, et al. [Diagnostic efficiency of hemoglobin A1c for newly diagnosed diabetes and prediabetes in community-based Chinese adults aged 40 years or older]. Diabetes Tech. 2014;16(12):853-7. Chinese.
63. Lyu J, Zhang W, Li W, Wang S, Zhang J. [Epidemic of chronic diseases and the related healthy lifestyle interventions in rural areas of Shandong province, China]. Bmc Pub Health. 2020;20(1):606. Chinese.
64. Wei M, Dong L, Wang F, Cui K, Ma D, Yang N, et al. The prevalence and control of type 2 diabetes mellitus in residents of a rural town, Shandong province, China. Diabetes Metab Syndr Obes. 2021;14:4505-12.
65. Yan X, Yang Y, Fu J, Li Q, Zhao Y. [Nutrition status and its risk factors among middle-aged and elderly residents with diabetes mellitus in urban and rural areas of Jiangxi province]. Prac Prev Med. 2018;25(7):784-7. Chinese.
66. Liu L. [Epidemiology survey and risk factors of diabetes mellitus in adults of Jiangxi province]. Nanchang: Nanchang University; 2016. Chinese.

67. Liu J, Zhu L, Li A, Luo W, Yan W, Ji L, et al. [Survey on Epidemic situation and influencing factors of diabetes mellitus among urban and rural residents in Jiangxi province]. Pract Prev Med. 2011;18(9):1637-8. Chinese.
68. Chen T, Li W, Zhong W, Lin X, Huang S, Ye Y. [Epidemiological characteristics of diabetes and influencing factors in adult in Fujian province]. Chronic Pathematol J. 2017;18(9):957-61. Chinese.
69. Ye Y, Lin S, Zhong W, Lin X, Lin X, Li X, et al. [Prevalence and influence factors of abnormal glucose metabolism among adult residents in Fujian province]. Chin J Pub Health. 2016;32(4):497-501. Chinese.
70. Lin L, Chen G, Zou X, Zhao J, Zhu F, Tu M, et al. [Diabetes, pre-diabetes and associated risks on Minnesota code-indicated major electrocardiogram abnormality among Chinese: a cross-sectional diabetic study in Fujian province, southeast China]. Obes Rev. 2009;10(4):420-30. Chinese.
71. Wang Q. [A study on prevalence of new-diagnosed diabetes mellitus and the establishment and evaluation for pre-diabetes screening model in Jiangsu province, China]. Nanjing: Southeast University; 2018. Chinese.
72. Xie W, Zhang J, Zhu Q, Dai Y. [The prevalence of diabetes among adult residents in Jiangsu province]. Jiangsu J Prev Med. 2019;30(05):522-5. Chinese.
73. Zhang YH, Ma WJ, Thomas GN, Xu YJ, Lao XQ, Xu XJ, et al. Diabetes and pre-diabetes as determined by glycated haemoglobin A1c and glucose levels in a developing southern Chinese population. PLoS One. 2012;7(5):e37260.
74. Li R, Lu W, Xu WH, Zhong WJ, Jiang QW, Li YY, et al. [Increasing prevalence of type 2 diabetes in Chinese adults in Shanghai]. Diabetes Care. 2012;35(5):1028-30. Chinese.
75. Zhang X. [Prevalence and control of diabetes in Shanghai]. Shanghai: The Second Military Medical University; 2018. Chinese.
76. Qin Y, Wang R, Ma X, Zhao Y, Lu J, Wu C, et al. [Prevalence, awareness, treatment and control of diabetes mellitus — a population based study in Shanghai, China]. Int J Env Res Pub He. 2016;13(5):512. Chinese.
77. Tang Z, Zeng F, Li Z, Zhou L. [Association and predictive value analysis for resting heart rate and diabetes mellitus on cardiovascular autonomic neuropathy in general population]. J Diabetes Res. 2014;2014:1-7. Chinese.
78. Yang Z, Xu B, Lu J, Tian X, Li M, Sun K, et al. Autonomic test by EZSCAN in the screening for prediabetes and diabetes. PLoS One. 2013;8(2):e56480.
79. Pei J, Li Y, Yang Y, Cheng M, Shi Y, Xu WH. [Prevalence trends and disease burden of diabetes and prediabetes in Chinese adults of Shanghai]. J Diabetes. 2023;15(7):583-96. Chinese.
80. Han S, Zhang P, Li J, Qi J, Liu J, Zhu S. [Analysis of prevalence and comorbidity factors of hypertension, diabetes mellitus and hyperlipidemia in 35 – 75 years old residents in Hubei province. J Applied Prev Med.

2019;25(5):356-60. Chinese.

81. Xu S, Ming J, Xing Y, Gao B, Yang C, Ji Q, et al. [Regional differences in diabetes prevalence and awareness between coastal and interior provinces in China: a population-based cross-sectional study]. BMC Public Health. 2013;13(1):299. Chinese.
82. Zhang X, Xu X, Dong T, Deng Y, Zeng J, Yi G, et al. [Prevalence and its risk factors of diabetes among adults in Sichuan, 2018]. Mod Prev Med. 2022;49(11):1931-6. Chinese.
83. Ding X, Mao D, Jiao Y, Chen L, Xu J. [Analysis of prevalence, mortality and disability-adjusted life year rate of diabetes in Chongqing city]. J Shanghai Jiaotong Univ (Med Sci). 2021;41(1):78-81. Chinese.
84. Duan M, Xi Y, Tian Q, Na B, Han K, Zhang X, et al. Prevalence, awareness, treatment and control of type 2 diabetes and its determinants among Mongolians in China: a cross-sectional analysis of IMAGINS 2015 – 2020. BMJ Open. 2022;12(11):e63893.
85. Wei W, Zhou M, Sha Q, Zhou S, Xu Z, Zhang S, et al. [Prevalence and risk factors of diabetes among residents in Qinghai province]. J Med Pest Control. 2013;29(1):62-6. Chinese.
86. Ma F, Zhou M, Yue J, Sha Q, Zhou S, Guo S. [Prevalence and awareness, treatment and control of major chronic diseases among adult residents in some areas of Qinghai province]. Chin J Prev Control Chronic Dis. 2015;23(11):854-6. Chinese.
87. Liu Y, Zhang X, He L, Wang S. [Prevalence and the relevant factors of type 2 diabetes mellitus among adults in Guizhou province in 2010 – 2012]. J Hygiene Res. 2019;48(5):723-7. Chinese.
88. Bai G, Yu Y, Shi H, Zha X. [Investigation on the prevalence of diabetes in Xizang residents]. World Latest Med Inf. 2019;19(37):271-5. Chinese.
89. Fu Y, Liu P, Wang X, Fu Z, Wang H, Wang S. [Analysis on diabetes mellitus prevalence and its risk factors in Hainan province]. Chin J Health Educat. 2017;33(11):967-71. Chinese.
90. Liu M, Yang S, Wang S, Li J, Ku F, Tai P, et al. [Prevalence of diabetes and associated factors in Hainan centenarians]. Chin J Epidemiol. 2021;42(1):68-72. Chinese.
